# The Influence of Glycans-Specific Bioconjugation on the FcγRI Binding and *In vivo* Performance of ^89^Zr-DFO-Pertuzumab

**DOI:** 10.7150/thno.39089

**Published:** 2020-01-01

**Authors:** Delphine Vivier, Kimberly Fung, Cindy Rodriguez, Pierre Adumeau, Gary A. Ulaner, Jason S. Lewis, Sai Kiran Sharma, Brian M. Zeglis

**Affiliations:** 1 Department of Chemistry, Hunter College, City University of New York, New York, NY, USA; 2 Ph.D. Program in Chemistry, The Graduate Center of the City University of New York, New York, NY, USA; 3 Department of Radiology, Memorial Sloan Kettering Cancer Center, New York, NY, USA; 4 Department of Radiology, Weill Cornell Medical College, Center, New York, NY, USA; 5 Program in Molecular Pharmacology, Memorial Sloan Kettering Cancer Center, New York, NY, USA

**Keywords:** ^89^Zr, pertuzumab, HER2, site-specific, glycans, immunoPET, radioimmunoconjugate

## Abstract

**Rationale**: The overwhelming majority of radioimmunoconjugates are produced *via* random conjugation methods predicated on attaching bifunctional chelators to the lysines of antibodies. However, this approach inevitably produces poorly defined and heterogeneous immunoconjugates because antibodies have several lysines distributed throughout their structure. To circumvent this issue, we have previously developed a chemoenzymatic bioconjugation strategy that site-specifically appends cargoes to the biantennary heavy chain glycans attached to C_H_2 domains of the immunoglobulin's Fc region. In the study at hand, we explore the effects of this approach to site-specific bioconjugation on the Fc receptor binding and *in vivo* behavior of radioimmunoconjugates.

**Methods**: We synthesized three desferrioxamine (DFO)-labeled immunoconjugates based on the HER2-targeting antibody pertuzumab: one using random bioconjugation methods (DFO-^nss^pertuzumab) and two using variants of our chemoenzymatic protocol (DFO-^ss^pertuzumab-EndoS and DFO-^ss^pertuzumab-βGal). Subsequently, we characterized these constructs and evaluated their ability to bind HER2, human FcγRI (huFcγRI), and mouse FcγRI (muFcγRI). After radiolabeling the immunoconjugates with zirconium-89, we conducted PET imaging and biodistribution studies in two different mouse models of HER2-expressing breast cancer.

**Results**: MALDI-ToF and SDS-PAGE analysis confirmed the site-specific nature of the bioconjugation, and flow cytometry and surface plasmon resonance (SPR) revealed that all three immunoconjugates bind HER2 as effectively as native pertuzumab. Critically, however, SPR experiments also illuminated that DFO-^ss^pertuzumab-EndoS possesses an attenuated binding affinity for huFcγRI (17.4 ± 0.3 nM) compared to native pertuzumab (4.7 ± 0.2 nM), DFO-^nss^pertuzumab (4.1 ± 0.1 nM), and DFO-^ss^pertuzumab-βGal (4.7 ± 0.2 nM). ImmunoPET and biodistribution experiments in athymic nude mice bearing HER2-expressing BT474 human breast cancer xenografts yielded no significant differences in the *in vivo* behavior of the radioimmunoconjugates. Yet experiments in tumor-bearing humanized NSG mice revealed that ^89^Zr-DFO-^ss^pertuzumab-EndoS produces higher activity concentrations in the tumor (111.8 ± 39.9 %ID/g) and lower activity concentrations in the liver and spleen (4.7 ± 0.8 %ID/g and 13.1 ± 4.0 %ID/g, respectively) than its non-site-specifically labeled cousin, a phenomenon we believe stems from the altered binding of the former to huFcγRI.

**Conclusion**: These data underscore that this approach to site-specific bioconjugation not only produces more homogeneous and well-defined radioimmunoconjugates than traditional methods but may also improve their *in vivo* performance in mouse models by reducing binding to FcγRI.

## Introduction

In recent years,^ 89^Zr-labeled antibodies have emerged as promising agents for the positron emission tomography (PET) imaging of cancer 1. Indeed, ^89^Zr is nearly ideally suited for immunoPET because its physical half-life (t_1/2_ = 3.3 days) aligns perfectly with the biological half-life of full-length antibodies 2-4. Recently, two radioimmunoconjugates targeting different epitopes of human epidermal growth factor receptor-2 (HER2) — ^89^Zr-DFO-trastuzumab and ^89^Zr-DFO-pertuzumab — have shown significant clinical potential in patients with HER2-positive breast cancer 5, 6. Yet despite the promise of ^89^Zr-labeled antibodies, the current synthetic methodologies used to create them are suboptimal at best. The majority of radioimmuno-conjugates are produced *via* random conjugation methods in which bifunctional chelators — in the case of ^89^Zr, desferrioxamine (DFO) — are attached to the lysines of the antibody 7. Yet because antibodies have several lysines distributed throughout their structure, this approach produces poorly defined and heterogeneous immunoconjugates that can suffer from impaired immunoreactivity.

A variety of different *site-specific* bioconjugation methods have been developed to circumvent these problems, including strategies based on thiol-reactive probes, peptide tags, and non-canonical amino acids 7-9. The benefits of site-specific bioconjugation are clear. Irrespective of the modification method, site-specifically modified immunoconjugates have been shown to be more homogeneous, better-defined, more reproducibly synthesized, and more effective *in vivo* than their randomly modified counterparts 7, 10-14. Moreover, several reports have shown that site-specifically modified radioimmunoconjugates exhibit improved *in vivo* behavior compared to randomly labeled analogues 15-20.

Over the last half-decade, our laboratory and others have worked to develop chemoenzymatic approaches to site-specific bioconjugation capable of selectively appending cargoes — including fluorophores, chelators, and toxins — to the heavy chain glycans on the C_H_2 domain of an antibody's Fc region 10, 14-16, 21-23. These biantennary sugar chains are particularly attractive sites for modification as they offer biochemically unique handles for manipulation and lie far from the antigen-binding domains of the immunoglobulin. Our strategy approach is predicated on three steps. First, one of two enzymes is used to truncate the glycans: either β-galactosidase (βGal, which removes the outermost monosaccharides) or endoglycosidase (EndoS, which hydrolyzes the chitobiose core of the glycans, leaving only the innermost residue). Second, a mutant, promiscuous galactosyltransferase [GalT-(Y289L)] is used to install azide-modified galactose residues (GalNAz) into the sugar chains. And finally, dibenzocyclooctyne-bearing cargoes are appended to the azide-bearing sugars *via* the strain-promoted azide-alkyne click (SPAAC) reaction. We have demonstrated that this strategy produces well-defined, more homogeneous immunoconjugates with excellent *in vivo* behavior using several model systems and a range of different payloads, and we are currently bringing this technology to the clinic 8, 15, 16.

In the work at hand, we set out to explore the influence of site-specific bioconjugation on the *in vivo* performance of radioimmunoconjugates. This investigation is fueled in large part by recent results from our laboratory that suggest that the truncation of the heavy chain glycans of radioimmunoconjugates can reduce their retention in healthy non-target organs and boost their accretion in tumor tissue 24, 25. We hypothesize that the root of this phenomenon lies in the conformational change that occurs upon the deglycosylation of the immunoglobulin. This change attenuates the binding of the immunoconjugate to FcγRI, an Fc receptor that is expressed on the surface of monocytes, macrophages, and tissue-resident macrophages in the liver 26, 27. It is important to note that we have focused on FcγRI in this investigation because it is the *only* member of the FcγR family that is able to bind monomeric IgGs; the others, FcγRII and FcγRIII, prefer to bind to immune-complexes 28. Due to its clinical relevance, we selected pertuzumab — a monoclonal antibody that targets the HER2 antigen over-expressed in 20-30% of breast cancers — for this proof-of-concept study 29, 30. In fact, the first-in-human clinical trial of ^89^Zr-DFO-pertuzumab in patients with HER2-positive breast cancer was conducted in 2017 5. Here, we synthesized three desferrioxamine-labeled pertuzumab immunoconjugates: one using traditional, random bioconjugation methods (DFO-^nss^pertuzumab) and two using our chemoenzymatic protocol (DFO-^ss^pertuzumab-EndoS and DFO-^ss^pertuzumab-βGal). Subsequently, we structurally characterized the trio of constructs, interrogated their ability to bind recombinant HER2 as well as human and mouse FcγRI, explored their *in vitro* behavior with HER2-positive BT474 human breast cancer cells, and evaluated their *in vivo* performance in two different mouse models of HER2-expressing human breast cancer, including one using humanized NSG (huNSG) mice. Taken together, the data clearly illustrate that site-specific bioconjugation not only produces more homogeneous and well-defined radioimmuno-conjugates than traditional methods but can also improve their *in vivo* performance in certain mouse models and — potentially — patient populations. While our laboratory and others have indeed previously noted the improved *in vivo* performance of radioimmunoconjugates that have been modified on the heavy chain glycans, this investigation represents the first exploration of why this may be the case 15-17.

## Methods

### Reagents and General Procedures

All chemicals, unless otherwise noted, were acquired from Sigma-Aldrich or Fisher Scientific and used as received without further purification. All water used was ultra-pure (>18.2 MΩcm^-1^), and dimethylsulfoxide was of molecular biology grade (>99.9%). DFO-^nss^pertuzumab, DFO-^ss^pertuzumab-βGal, and DFO-^ss^pertuzumab-EndoS were prepared according to published protocols and characterized via SDS-PAGE, MALDI-ToF mass spectrometry, and ELISA according to previously reported methods 15, 16. [^89^Zr]Zr(oxalate) was produced and purified via the ^89^Y(p,n)^89^Zr reaction at Memorial Sloan Kettering Cancer Center as previously described. Activity measurements were made using a CRC-15R Dose Calibrator (Capintec, Inc.), and experimental samples were counted on an Automatic Wizard^2^ γ-counter (PerkinElmer, Inc.). The radiolabeling of the pertuzumab immunoconjugates with [^89^Zr]Zr^4+^ was performed according to published procedures and monitored using silica-impregnated instant thin-layer chromatography (iTLC) paper (Pall Corp.) on an AR-2000 radio-TLC plate reader (Bioscan, Inc.). Mice were implanted with HER2-positive BT474 human breast cancer xenografts as previously reported, and PET imaging and acute biodistribution experiments were performed according to published protocols approved by the Institutional Animal Care and Use Committees of Hunter College, Weill Cornell Medical College, and Memorial Sloan Kettering Cancer Center 15, 16.

### Surface Plasmon Resonance

Binding affinities (K_D_) and kinetic constants (k_a_ and k_d_) for pertuzumab as well as the three DFO-modified immunoconjugates — DFO-^nss^pertuzumab, DFO-^ss^pertuzumab-βGal and DFO-^ss^pertuzumab-EndoS — were determined *via* surface plasmon resonance (SPR) on a Biacore T200 instrument (GE Healthcare).

First, in order to characterize the impact of bioconjugation on the immunoreactivity of pertuzumab for its cognate antigen, SPR experiments (n = 3 per construct) with HER2 were performed. To this end, pertuzumab or its DFO-modified immunoconjugates were captured as the ligand on a Protein A sensor chip (29-1275-56, GE Healthcare) by diluting the IgG to a concentration of 1 μg/mL in HBS-EP+ buffer (BR100188, GE Healthcare) and injecting it over a Series S protein A sensor chip for 30 s at a flow rate of 5 μL/min. Next, purified recombinant human HER2 protein (HE2-H822R Acro Biosystems) was used as the analyte and flowed over the functionalized sensor chip. The binding kinetics were evaluated over a range of HER2 concentrations in HBS-EP+ buffer (25 nM, 12.5 nM, 6.25 nM, 3.13 nM, 1.56 nM, 0.78 nM, 0.39 nM and 0.195 nM), with each concentration injected for 5 minutes at a flow rate of 5 µL/min to facilitate binding with the immunoconjugate captured on the sensor chip. The dissociation phase was evaluated by allowing the binding buffer (HBS-EP+) to flow (5 µL/min) over the sensor chip for 900 s for the highest analyte concentration (25 nM) and 300 s for all other concentrations. The regeneration buffer (10 mM Glycine-HCl pH 1.5) was then passed over the chip surface for 1 min at a flow rate of 5 µL/min to achieve complete dissociation of the captured antibody and any remaining immunocomplexes. And finally, HBS-EP+ buffer was flowed over the chip for 2 min at 5 µL/min to stabilize the protein A chip surface prior to the injection of the next sample.

Next, to investigate the impact of bioconjugation and deglycosylation on the interaction between the immunoconjugates and Fc receptors, we turned to SPR experiments with the human and murine variants of the high affinity Fc-receptor: FcγRI. With respect to the former, a histidine-tagged variant of recombinant human FcγRI (500238; NovoPro Labs) was used as the ligand. A Series S sensor chip CM5 (29401988; GE Healthcare) was functionalized with an anti-histidine antibody using components from the His-capture kit (28995056; GE Healthcare) and the amine coupling kit (BR-1000-50; GE Healthcare) following the standard procedure prescribed by the application wizard on the Biacore T200. After the chip was functionalized, a 0.8 nM solution of huFcR1 in running buffer (HBS-P+ buffer containing 50 μM EDTA) was injected over flow cell 2 for 60 s at a flow rate of 10 μL/min. Subsequently, high performance injections of various concentrations of pertuzumab or its DFO-modified conjugates (100 nM, 50 nM, 25 nM, 12.5 nM, 6.25 nM, 3.13 nM, 1.56 nM, and 0.78 nM) were performed over flow cells 1 and 2 for 5 min at a flow-rate of 30 μL/min. The dissociation of the analyte was evaluated by allowing the running buffer to flow over the chip surface for 5 min. Finally, the chip surface was regenerated using a 60 s injection of 10 mM Glycine-HCl (His-capture kit) at a flow-rate of 30 μL/min, followed by an extra wash with the running buffer. A similar — though slightly modified — experimental set up was used to evaluate the interaction between the immunoconjugates and murine FcγRI (muFcγRI). Here, a 2.4 nM solution of histidine-tagged mouse FcγRI (2074-FC; R&D Systems) was captured on the same series S CM5 sensor chip as used above. Based on preliminary indications suggesting rapid on- and off-rates, the association and dissociation phases for all of the pertuzumab-muFcγRI interactions were evaluated over a shorter time window: 120 s. The Biacore T200 evaluation software was used to analyze the kinetic data. A 1:1 fit (RI set to 0) was used to derive kinetic constants for the interactions between the immunoconjugates and both HER2 and huFcγRI. In contrast, the rapid on- and off-rates observed in case of muFcγRI warranted analysis using steady-state kinetics.

### Statistical analysis

Data were analyzed by the unpaired, two-tailed Student's t test. Differences at the 95% confidence level (p < 0.05) were considered to be statistically significant.

## Results

### Model System, Synthesis, and Characterization

Assembling the three components of the model system for this proof-of-concept study was fairly straightforward. Zirconium-89 (t_1/2_ ~ 3.3 d) is the radionuclidic gold standard for immunoPET, and desferrioxamine (DFO) remains the only clinically-employed chelator for the radiometal (though several other options are on the cusp of being tested in the clinic) 1. The identity of the antibody presented a wider array of options, but the HER2-targeting pertuzumab emerged as the ideal candidate. Indeed, the recent clinical translation of ^89^Zr-DFO-pertuzumab at Memorial Sloan Kettering Cancer Center not only illustrated the clinical promise of the radiotracer but also — and just as importantly — paved the way for future clinical comparisons with a site-specifically labeled variant of the radioimmunoconjugate.

**Figure 1:**
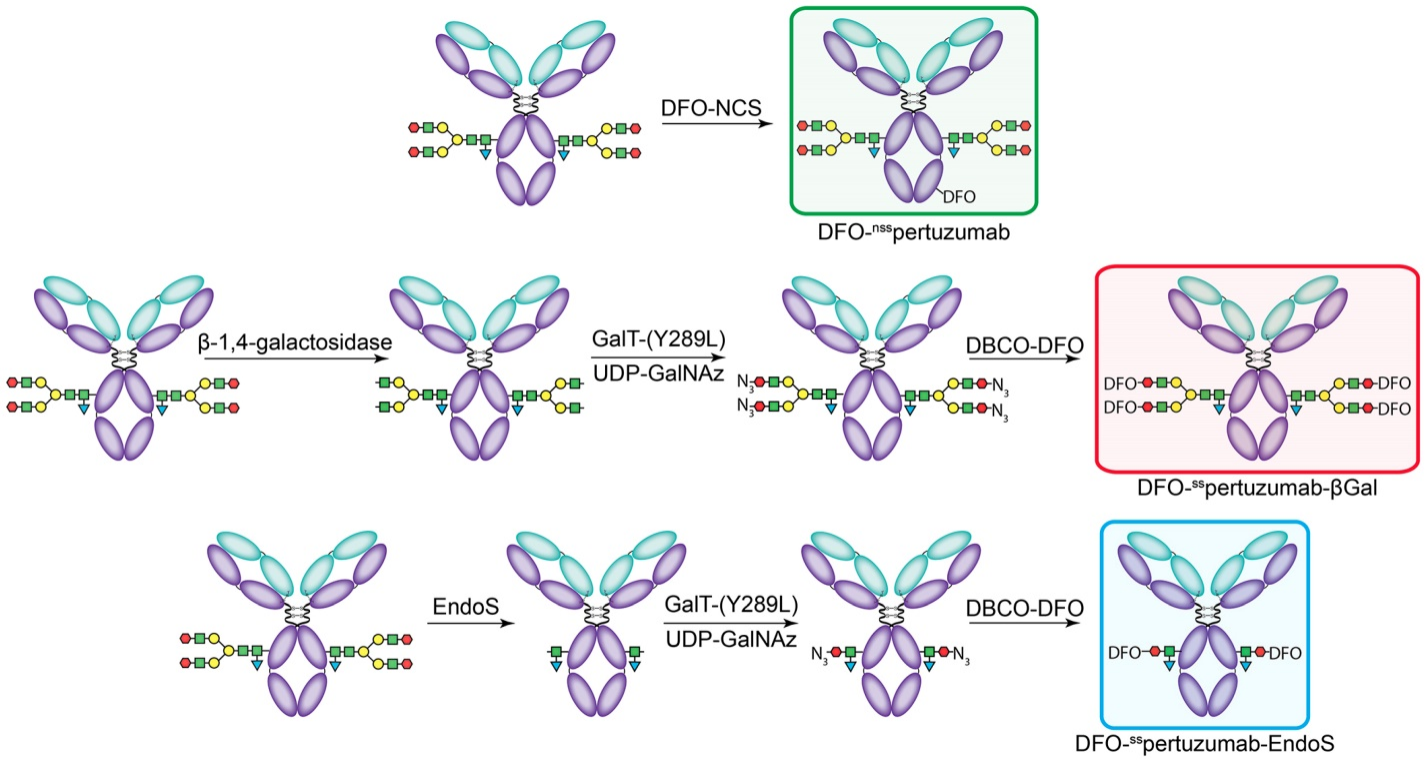
The construction of the pertuzumab immunoconjugates: DFO-^nss^pertuzumab (top), DFO-^ss^pertuzumab-βGal (middle), and DFO-^ss^pertuzumab-EndoS (bottom).

The non-site-specifically modified immunoconjugate — DFO-^nss^pertuzumab — was prepared according to published procedures *via* the random conjugation of an isothiocyanate-bearing variant of DFO (*p*-SCN-Bn-DFO) to the lysines of the antibody (*Figure 1A*) 31. MALDI-ToF analysis of the resulting immunoconjugate - DFO-^nss^pertuzumab - revealed an average of 1.4 ± 0.4 DFO attached *per* antibody (*Supplemental Figures S1-S6 and Table S1*). The site-specifically modified immunoconjugates were synthesized using a chemoenzymatic method developed in our laboratory 15, 16. Briefly, pertuzumab was first treated with one of two enzymes in order to expose terminal *N*-acetylglucosamine residues: β-1,4-galactosidase, which removes the terminal galactose residues of the glycans, or EndoS, which hydrolyzes the chitobiose core of the asparagine-linked glycans (*Figure 1B-C*). The resulting constructs were then incubated with the promiscuous galactosyltransferase Gal-T1(Y289L) and the monosaccharide UDP-GalNaz to incorporate azides into the remaining glycans. Finally, the chelator desferrioxamine (DFO) was introduced *via* the strain promoted alkyne-azide cycloaddition between dibenzocyclooctyne (DBCO)-DFO and the azide-bearing glycans, ultimately providing DFO-^ss^pertuzumab-βGal or DFO-^ss^pertuzumab-EndoS. Sodium dodecyl sulfate-polyacrylamine gel electrophoresis (SDS-PAGE) confirmed the site-specificity of the modification (*Supplemental Figure S7*), size exclusion chromatography illustrated that none of the immunoconjugates forms aggregates (*Supplemental Figures S8-S11*), and MALDI-ToF mass spectrometry revealed degrees of labeling of 2.6 ± 0.1 DFO/mAb for DFO-^ss^pertuzumab-βGal and 1.3 ± 0.2 DFO/mAb for DFO-^ss^pertuzumab-EndoS (*Table S1*).

### *In vitro* characterization

The ability of the immunoconjugates to bind HER2 was first interrogated via SPR assays. These experiments revealed that native pertuzumab, DFO-^nss^pertuzumab, DFO-^ss^pertuzumab-βGal, and DFO-^ss^pertuzumab-EndoS all exhibit nearly identical values for K_D_ (~0.14-0.16 nM), k_a_ (1.9-2.1 × 10^5^ M^-1^s^-1^), and k_d_ (2.7-3.1 × 10^-5^ M^-1^) (*Table 1 and Supplemental Figure S12*). These results were confirmed with flow cytometry experiments using HER2-expressing BT474 human breast cancer cells (*Supplemental Figure S13*). Taken together, these data suggest that the switch to site-specific bioconjugation does not perturb the ability of the immunoconjugates to bind their target antigen.

**Figure 2:**
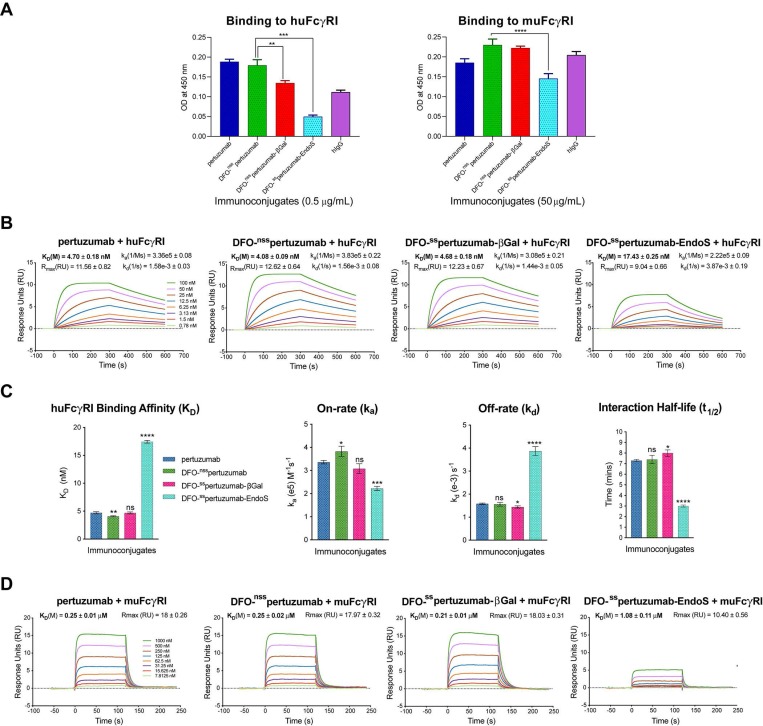
** Comparative SPR and ELISA analysis of the interaction between the pertuzumab immunoconjugates and recombinant human and mouse FcγRI. (A)** ELISA data demonstrating the binding of the immunoconjugates to huFcγRI (10 μg/mL; left) and muFcγRI (10 μg/mL; right); **(B)** Sensorgrams showing robust dose-response curves and kinetic profiles for the binding of various concentrations of native pertuzumab and the DFO-bearing immunoconjugates to huFcγRI; **(C)** Bar graphs demonstrating the correlation between deglycosylation and binding affinity (K_D_), on-rate (k_a_), off-rate (k_d_) and half-life (t_1/2_). The binding affinity, kinetic constants and half-lives for each of the DFO-bearing immunoconjugates were compared with those obtained for unmodified pertuzumab; **(D)** Sensorgrams showing robust dose-response curves and kinetic profiles for the binding of various concentrations of native pertuzumab and the DFO-bearing immunoconjugates to muFcγRI. Statistically significant relationships are indicated with asterisks. * = p < 0.05, ** = p < 0.005, *** = p < 0.0005, **** = p < 0.00001.

In light of our recent work on the interplay between the glycosylation, Fc receptor binding, and *in vivo* performance of radioimmunoconjugates (see *Discussion*), we also probed the binding of the three immunoconjugates to murine and human FcγRI *via* ELISA and SPR. The ELISA data illustrate that DFO-^ss^pertuzumab-EndoS exhibits attenuated binding to huFcγRI compared to DFO-^nss^pertuzumab (*Figure 2A*). Somewhat surprisingly, DFO-^ss^pertuzumab-βGal also exhibits decreased binding to huFcγRI, though not to the same degree as its more truncated counterpart. These data are generally reinforced by the ELISA experiments with muFcγRI, though a higher concentration of immunoconjugate was needed to generate meaningful data due to the lower affinity of the murine receptor for the human IgGs. Here, DFO-^ss^pertuzumab-EndoS again displays reduced binding to muFcγRI compared to DFO-^nss^pertuzumab, while DFO-^ss^pertuzumab-βGal occupies a middle ground.

Broadly speaking, the SPR data confirm the ELISA findings (*Figure 2B-D and Table 2*) and are in strong agreement with our recent findings on deglycosylated radioimmunoconjugates 24. With respect to huFcγRI, it is clear that the truncation of the heavy glycans exerts a strong influence on binding. More specifically, native pertuzumab (4.7 ± 0.2 × 10^-9^ M), DFO-^nss^pertuzumab (4.1 ± 0.1 × 10^-9^ M), and DFO-^ss^pertuzumab-βGal (4.7 ± 0.2 × 10^-9^ M) exhibit similar binding affinities for the receptor, while that of DFO-^ss^pertuzumab-EndoS is weaker: 17.4 ± 0.3 × 10^-9^ M (*Table 2*). A similar trend is seen in the kinetic parameters. Here, the EndoS-modified immunoconjugate has a slower on-rate and an accelerated off-rate compared to the other immunoconjugates. These differences translate to a shorter interaction half-time for DFO-^ss^pertuzumab-EndoS (3.0 ± 0.1 min) compared to native pertuzumab (7.3 ± 0.1 min), DFO-^nss^pertuzumab (7.4 ± 0.4 min), and DFO-^ss^pertuzumab-βGal (8.0 ± 0.3 min).

The parallel examination of the interactions between the pertuzumab immunoconjugates and murine FcγRI revealed a similar overall trend but also a few key differences. While all four immunoconjugates displayed 50-fold lower binding constants for muFcγRI compared to huFcγRI, unmodified pertuzumab (2.5 ± 0.1 × 10^-7^ M), DFO-^nss^pertuzumab (2.5 ± 0.2 × 10^-7^ M), and DFO-^ss^pertuzumab-βGal (2.1 ± 0.1 × 10^-7^ M) still retained higher affinities than DFO-^ss^pertuzumab-EndoS (1.1 ± 0.1 × 10^-6^ M). Furthermore, the quartet of immunoconjugates displayed rapid on- and off-rates for binding to muFcγRI, thereby necessitating the analysis of the interaction using steady-state kinetics and abrogating the determination of kinetic parameters.

### Radiolabeling

The immunoconjugates were subsequently radiolabeled with ^89^Zr using standard protocols, producing a trio of radioimmunoconjugates in >95% radiochemical yield and >99% radiochemical purity with similar specific activities of 96.2 ± 1.1 MBq/mg (14.4 ± 0.2 GBq/μmol) for ^89^Zr-DFO-^nss^pertuzumab, 96.2 ± 1.5 MBq/mg (14.4 ± 0.2 GBq/μmol) for ^89^Zr-DFO-^ss^pertuzumab-βGal, and 97.7 ± 0.7 MBq/mg (14.7 ± 0.1 GBq/μmol) for ^89^Zr-DFO-^ss^pertuzumab-EndoS. A subsequent stability study in human serum at 37 °C revealed that over the course of a week, the three radioimmunoconjugates demonstrated >85% stability (Supplemental *Figure S14*). Similarly, a bead-based HER2 binding assay (see *Supplemental Methods*) yielded nearly identical immunoreactivities for the radioimmunoconjugates: 79 ± 4%, 92 ± 2%, 88 ± 3% for ^89^Zr-DFO-^nss^pertuzumab, ^89^Zr-DFO-^ss^pertuzumab-βGal, and ^89^Zr-DFO-^ss^pertuzumab-EndoS, respectively.

### *In vivo* behavior

**Figure 3:**
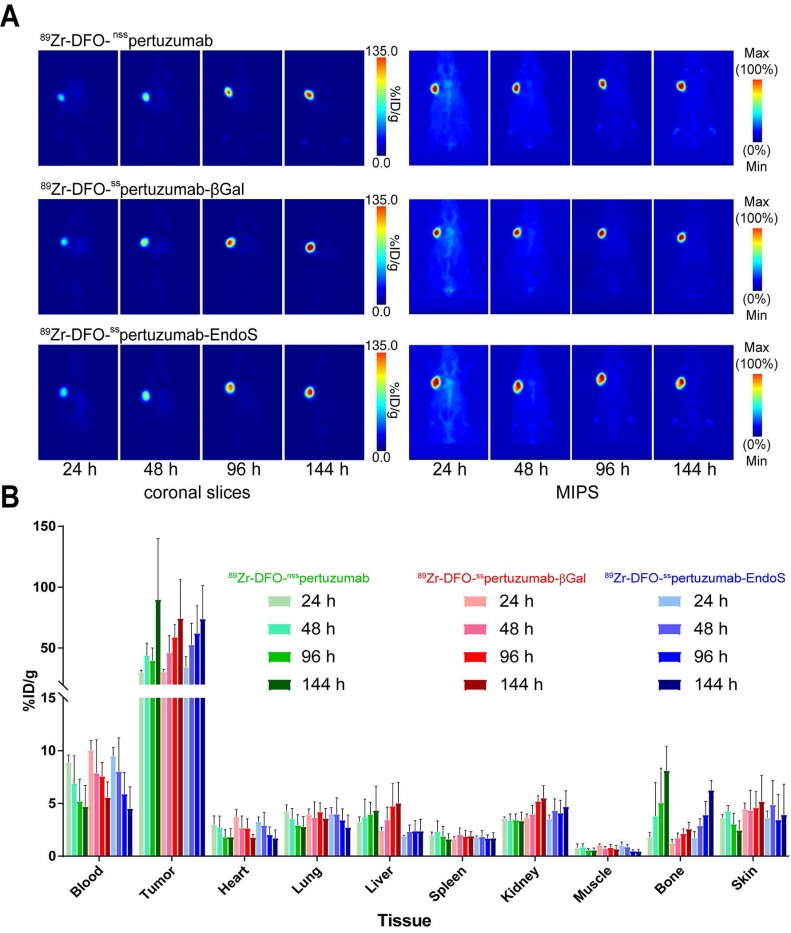
** (A)** Planar (left) and maximum intensity projection (MIP, right) PET images of athymic nude mice bearing subcutaneous BT474 xenografts injected with the three ^89^Zr-DFO-pertuzumab radioimmunoconjugates (179 - 192 μCi, 6.6 - 7.1 MBq, 85 - 92 μg, in 200 μL 0.9% sterile saline). **(B)** Biodistribution data for athymic nude mice bearing HER2-expressing BT474 xenografts injected with ^89^Zr-DFO-^nss^pertuzumab**,** ^89^Zr-DFO-^ss^pertuzumab-EndoS, or ^89^Zr-DFO-^ss^pertuzumab-βGal (lateral tail vein injection, 15 - 20 µCi, 0.56 - 0.74 MBq, 6 - 9 µg).

In order to compare the *in vivo* performance of the radioimmunoconjugates, PET imaging and biodistributions experiments were first conducted in athymic nude mice bearing subcutaneous HER2-expressing BT474 xenografts. PET images were collected at 24, 48, 96 and 144 h after the intravenous administration of each radioimmunoconjugate (200 μCi, 7.4 MBq, 70-80 μg). As early as 24 h post injection (p.i.) the tumors could easily be delineated in the images acquired with all three radioimmuno-conjugates (*Figure 3A*). Over the course of 6 days, the activity concentrations in the tumor increased dramatically, while the uptake in the blood decreased apace. The biodistribution data confirm the observations made *via* PET: as the experiment progressed, activity concentrations in the tumor increased, ultimately producing excellent tumor-to-healthy organ activity concentration ratios (*Figure 3B; Supplemental Tables S2 and S3*). Critically, very few qualitative or quantitative differences were found between the images and uptake values obtained with each of the radioimmunoconjugates.

**Figure 4:**
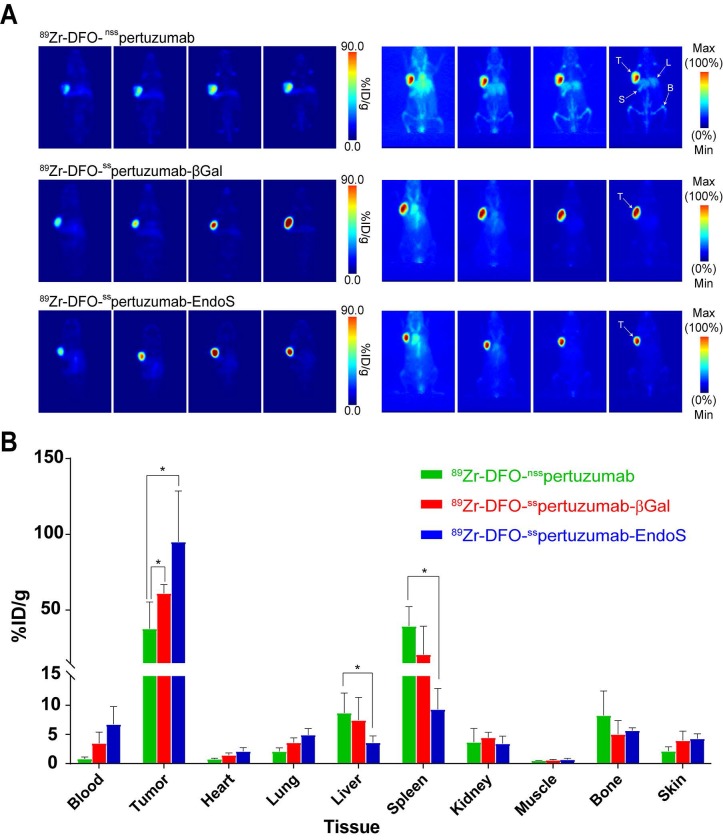
** (A)** Planar (left) and maximum intensity projection (MIP, right) PET images of huNSG mice bearing subcutaneous BT474 xenografts collected between 24 and 144 h after the administration of the three radioimmunoconjugates (209 - 218 μCi, 7.7 - 8.1 MBq, 80 - 83 μg, in 200 μl 0.9% sterile saline); **(B)** Biodistribution data for ^89^Zr-DFO-*^nss^*pertuzumab**,** ^89^Zr-DFO-^ss^pertuzumab*-*βGal, and ^89^Zr-DFO-^ss^pertuzumab-EndoS 144 hours following administration in huNSG mice bearing subcutaneous HER2-expressing BT474 xenografts. T = tumor; L = liver; S = spleen; B = bone. * = p < 0.05.

As we have noted, the initial *in vivo* experiments did not reveal any differences between the performances of the three radioimmunoconjugates. However, pertuzumab is a humanized antibody, athymic nude mice express murine (rather than human) FcγRI, and — as we have seen — the affinity of the pertuzumab-based immunoconjugates for muFcγRI is rather low. In order to investigate this phenomenon in a more appropriate mouse model, we turned to humanized NOD scid gamma (huNSG) mice. HuNSG mice are NSG mice that were sub-lethally irradiated 3 weeks after birth and then reconstituted with human hematopoietic stem cells in order to facilitate the expression of a functional human immune system, including human NK cells, dendritic cells, T cells, B cells, and monocytes 32-35. PET imaging was performed using huNSG mice bearing subcutaneous HER2-expressing BT474 xenografts (*Figure 4A*). The images reveal stark differences between the randomly-labeled radioimmunconjugate and the two site-specifically labeled variants. To wit, ^89^Zr-DFO-^nss^pertuzumab produces lower tumor uptake and higher activity concentrations in healthy organs — specifically, the liver, spleen, and bones — than ^89^Zr-DFO-^ss^pertuzumab-EndoS and ^89^Zr-DFO-^nss^pertuzumab-βGal. The biodistribution data at 120 h p.i. tell a slightly more nuanced story (*Figure 4B; Supplemental Tables S4 and S5*). ^89^Zr-DFO-^ss^pertuzumab-EndoS produces higher activity concentrations in the tumor compared to ^89^Zr-DFO-^nss^pertuzumab (111.8 ± 39.9 %ID/g *vs*. 46.1 ± 16.5 %ID/g; p < 0.05) as well as lower uptake in the liver (4.7 ± 0.8 %ID/g *vs.* 10.3 ± 2.7 %ID/g; p < 0.05) and spleen (13.1 ± 4.0 %ID/g 44.8 ± 7.9 %ID/g; p < 0.05). Interestingly, the biodistribution of ^89^Zr-DFO-^nss^pertuzumab-βGal presents an intermediate case. Its activity concentration in the tumor (77.5 ± 12.6 %ID/g) is greater than that of ^89^Zr-DFO-^nss^pertuzumab, though statistically significant differences in the uptake of the two radioimmunoconjugates in the liver and spleen were not observed.

## Discussion

While the advent of radioimmunoconjugates for nuclear imaging and therapy has coincided with the rise of 'precision medicine', the strategies used to create these tools have long remained surprisingly imprecise. The random conjugation of bifunctional chelators to the lysine residues of immunoglobulins is an undeniably simple approach that nonetheless produces poorly defined and heterogeneous immunoconjugates and risks compromising their bioactivity. The chemoenzymatic bioconjugation methodology that we have developed over the last five years offers a facile — though admittedly slightly more complex — route to better defined, more homogeneous, and highly immunoreactive agents. The key, of course, lies in leveraging the heavy chain glycans, as these biantennary sugar chains provide a handle for selective manipulation, a limited number (*i.e.* 2 or 4) of conjugation sites, and distance from the immunoglobulin's antigen-binding domains.

In the case at hand, the very nature of the modification strategy dictates that both DFO-^ss^pertuzumab-EndoS and DFO-^nss^pertuzumab-βGal are better defined and more homogeneous than DFO-^nss^pertuzumab. Yet beyond this fundamental — and important — difference, the three immunoconjugates proved very similar according to the metrics upon which immunoconjugates are traditionally evaluated. SPR assays revealed that each boasts sub-nanomolar K_D_ values for HER2 (0.14 nM - 0.16 nM) that are essentially identical to that of the parent antibody, findings that were reinforced by FACS with HER2-expressing BT474 cells. The similarities continued upon radiolabeling: ^89^Zr-DFO-^nss^pertuzumab,^ 89^Zr-DFO-^ss^pertuzumab-EndoS, and DFO-^nss^pertuzumab-βGal were produced in similar radiochemical yields and radiochemical purities and exhibited nearly identical specific activities, stabilities, and immunoreactivities. Clearly, this approach to bioconjugation provides structural benefits without any attendant costs when it comes to antigen binding and radiosynthesis.

Things become more interesting — and more complicated — when we consider that the manipulation of the heavy chain glycans is not necessarily benign. As we have noted, the heavy chain glycans play an important role in the structure of immunoglobulins and, as a result, their ability to bind Fcγ receptors, most notably FcγRI. We recently explored the interplay between the truncation of the heavy chain glycans, FcγRI binding, and the *in vivo* behavior of *randomly-modified* radioimmuno-conjugates using a series of^ 89^Zr-labeled variants of the HER2-targeting antibody trastuzumab 24. More specifically, we observed that the truncation (via EndoS) or removal (via PNGaseF) of the heavy chain glycans of ^89^Zr-DFO-trastuzumab attenuated the binding of the radioimmunoconjugates to FcγRI and improved *in vivo* performance in tumor-bearing NSG and huNSG mice. Critically, our results are corroborated by the work of others on antibody-drug conjugates and fluorophore-modified antibodies 36, 37.

This investigation extends our previous work to include a systematic evaluation of immunoconjugates created using both chemoenzymatic methods of bioconjugation. Both of the site-specifically modified immunoconjugates in this study have altered glycans: those of DFO-^ss^pertuzumab-βGal have simply been modified by the addition (*via* SPAAC) of DFO, while those of DFO-^ss^pertuzumab-EndoS have been truncated *and* modified with the chelator. These two bioconjugation pathways had little influence on the ability of the immunoconjugates to bind HER2 (*vide supra*), but they did impact their ability to bind human and murine FcγRI. Both the ELISA and SPR data illustrate that DFO-^ss^pertuzumab-EndoS exhibits attenuated binding to huFcγRI compared to native pertuzumab and DFO-^nss^pertuzumab, with the SPR data revealing both thermodynamic *and* kinetic effects. While the immunoconjugates displayed lower binding affinities for muFcγRI across the board, DFO-^ss^pertuzumab-EndoS again exhibited attenuated binding compared to the trio of fully glycosylated analogues according to both SPR and ELISA. Things become more complicated with DFO-^ss^pertuzumab-βGal. In this case, both the ELISA and the SPR data agree that the immunoconjugate exhibits similar affinity to native pertuzumab and DFO-^nss^pertuzumab for muFcγRI. In the case of huFcγRI, however, the ELISA and SPR data diverge slightly. The ELISA data shows that DFO-^ss^pertuzumab-βGal displays a decreased affinity for huFcγRI compared to DFO-^nss^pertuzumab and DFO-^ss^pertuzumab-EndoS, while the SPR data suggest that its binding to huFcγRI is nearly identical to that of the two fully glycosylated analogues. These slight deviations may simply be the result of inherent differences between the assays, especially since some small differences in the kinetic parameters of the binding of DFO-^ss^pertuzumab-βGal to huFcγRI *can* be seen in the SPR data.

With the relationship between glycans-specific bioconjugation and FcγRI binding largely established, the next step was to investigate whether these differences translate into changes in *in vivo* performance. As we have discussed (see *Results*), the three radioimmunoconjugates — ^89^Zr-DFO-^nss^pertuzumab, ^89^Zr-DFO-^ss^pertuzumab-EndoS, ^89^Zr-DFO-^ss^pertuzumab-βGal — exhibited all but identical behavior in athymic nude mice, suggesting that neither the increased homogeneity nor the attenuated FcγRI binding conferred by site-specific bioconjugation translates to significant *in vivo* improvements in this particular mouse model. These results are similar to those observed in our previous work and likely stem from the presence of endogenous murine IgG, the model's expression of muFcγRI (rather than huFcγRI), and the immunoconjugates' lower binding affinities for muFcγRI compared to huFcγRI 24, 38, 39.

The results in the huNSG mice — which, of course, express huFcγRI rather than muFcγRI — tell a more interesting story. In this model, ^89^Zr-DFO-^ss^pertuzumab-EndoS exhibited lower activity concentrations in the liver and spleen and higher uptake in the tumor than ^89^Zr-DFO-^nss^pertuzumab, results consistent with reductions in the interaction between ^89^Zr-DFO-^ss^pertuzumab-EndoS and FcγRI receptors expressed by monocytes, macrophages, and tissue-resident macrophages in the liver and spleen. The more subtle differences between the tumoral, hepatic, and splenic activity concentrations of ^89^Zr-DFO-^ss^pertuzumab-βGal and ^89^Zr-DFO-^nss^pertuzumab suggest an intermediate case that may relate to the less pronounced differences between the FcγRI binding of the two radioimmunoconjugates or to the increased homogeneity of the former relative to the latter. In light of these data, however, it is important to reinforce that huNSG mice are far from a flawless animal model. To wit, while they do express endogenous IgG (202 ± 67 μg/mL), the lack of class switching from the isotype IgM to the isotype IgG results in significantly lower titers than in immunocompetent strains and most human patients. This phenomenon can most likely be linked to the lack of B-cell maturation in huNSG mice due to the absence of follicular dendritic cells, germinal centers, and functional lymph nodes 32, 33, 40. As a result, huNSG mice may not provide the most accurate reflections of the behavior of these 'immune-silent' radioimmunoconjugates in immunocompetent patients. To remedy this limitation, we are currently working with collaborators to build and utilize murine models of disease that offer even more clinically relevant FcγR expression profiles and recapitulations of the human immune environment.

## Conclusion

In the preceding pages, we have demonstrated that the site-specific bioconjugation of antibodies *via* the manipulation of the heavy chain glycans not only facilitates the reproducible synthesis of better-defined and more homogenous immunoconjugates but can also provide radiotracers with improved *in vivo* performance. More specifically, compared to a traditional approach to antibody modification, the site-specific bioconjugation of pertuzumab using EndoS produces well-defined and highly immunoreactive immunoconjugates with attenuated binding to murine and human FcγRI. This approach improves *in vivo* behavior in certain immunocompromised mouse models (*i.e.* huNSG), a result that can have important implications for preclinical research. In retrospect, the lack of any fundamental differences in the antigen-binding behavior of the radioimmunoconjugates upon site-specific bioconjugation was actually advantageous, as it allowed us to parse between changes in biodistribution due to differences in immunoreactivity and changes in biodistribution due to differences in FcγRI binding (*vide infra*). It remains unclear whether this phenomenon could prove beneficial in the context of clinical immunoPET, though we hypothesize that it is most likely to improve imaging results in immunosuppressed patients with low titers of endogenous IgG. Moving forward, we plan to interrogate the utility of this phenomenon in the context of radioimmuno-therapeutics and antibody-drug conjugates. Furthermore — and undeniably more importantly — an upcoming clinical trial comparing the *in vivo* performance of ^89^Zr-DFO-^nss^pertuzumab and ^89^Zr-DFO-^ss^pertuzumab-EndoS will certainly help resolve the question of the applicability of this work to human immunoPET.

## Supplementary Material

Supplementary materials and methods, figures and tables.

## Figures and Tables

**Table 1:**
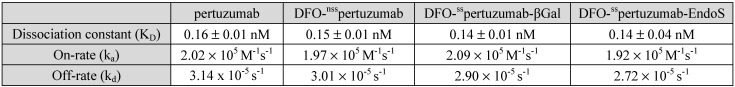
SPR-derived binding parameters for the pertuzumab immunoconjugates and HER2.

**Table 2:**
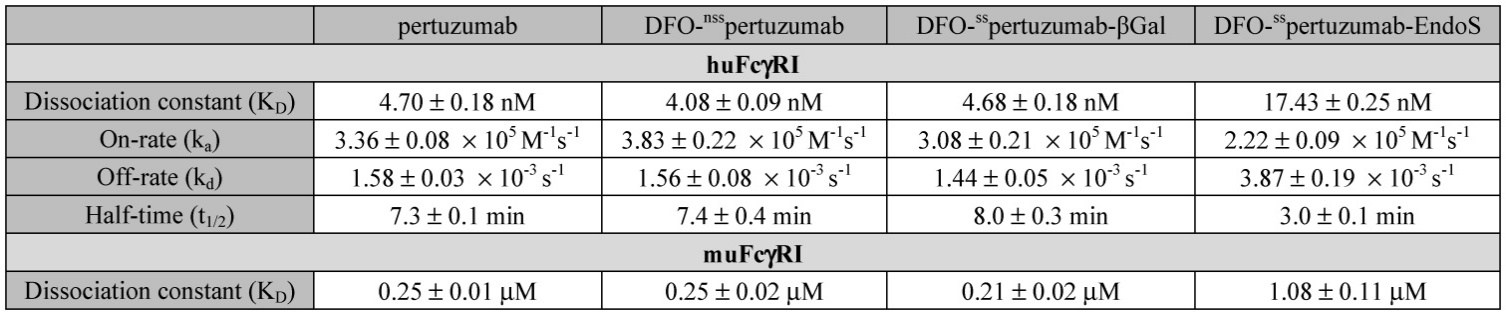
SPR-derived binding parameters for the pertuzumab immunoconjugates and human and murine FcγRI.

## Abbreviations

PET: positron emission tomography
DFO: desferrioxamine
%ID/g: percent injected dose per gram
p.i.: post-injection
NCS: isothiocyanate
DBCO: dibenzocyclooctyne
