# Supplementary material for: The Influence of Glycans-Specific Bioconjugation on the FcγRI Binding and In vivo Performance of 89Zr-DFO-Pertuzumab
Source: Theranostics. 2020 Jan 1;10(4):1746–57. doi: 10.7150/thno.39089 (PMC6993239; doi:10.7150/thno.39089)
Supplement: Supplementary file 1 — Supplementary materials and methods, figures and tables. [file thnov10p1746s1.pdf]

Supporting Information

The Influence of Glycans-Specific Bioconjugation on the FcγRI Binding and In Vivo Performance of ^{89}Zr-DFO-Pertuzumab

Delphine Vivier[1,†], Kimberly Fung[1,2,†], Cindy Rodriguez[1,2], Pierre Adumeau[1], Gary A. Ulaner[3,4], Jason S. Lewis[3,4,5], Sai Kiran Sharma[3], Brian M. Zeglis[1,2,3*]*

[1] Department of Chemistry, Hunter College, City University of New York, New York, NY, USA

[2] Ph.D. Program in Chemistry, The Graduate Center of the City University of New York, New York, NY, USA

[3]Department of Radiology, Memorial Sloan Kettering Cancer Center, New York, NY, USA

[4]Department of Radiology, Weill Cornell Medical College, Center, New York, NY, USA

[5]Program in Molecular Pharmacology, Memorial Sloan Kettering Cancer Center, New York, NY, USA

[†]These two authors contributed equally to this work.

Co-Corresponding Authors:

Brian M. Zeglis: 413 East 69th Street, New York, NY, 10021. Phone: 212-896-0433. E-mail: bz102@hunter.cuny.edu

Sai Kiran Sharma: 1285 York Avenue, New York, NY, 10025. Phone: 212-896-0484. E-mail: sharmas@mskcc.org

TABLE OF CONTENTS

Supplemental Materials and Methods 3
SDS-PAGE Analysis 3
Size Exclusion Chromatography 3
Flow Cytometry 3
FcγRI Binding ELISA 4
Radiolabeling with ^{89}Zr 4
Bead-Based Immunoreactivity Assay 5
Radioimmunoconjugate Stability Assays 5
PET Imaging 6
Acute Biodistribution Experiments 6
Supplemental Figures and Figure Captions 7
Supplemental Tables and Table Legends 16

SUPPLEMENTAL MATERIALS AND METHODS

SDS-PAGE Analysis

The purified constructs − pertuzumab, DFO-^{nss}pertuzumab, DFO-^{ss}pertuzumab-βGal, and DFO-^{ss}pertuzumab-EndoS − were characterized via sodium dodecyl sulfate-polyacrylamide gel electrophoresis (SDS-PAGE). Briefly, 2 µg antibody (0.85 µL of a 5.89 mg/mL stock) were combined with 31.65 µL H_2O, 5 µL 500 mM dithiothreitol (NuPAGE® 10X Sample Reducing Agent, Life Technologies), and 12.5 µL 4X electrophoresis buffer (NuPAGE® LDS Sample Buffer, Thermo Fisher, Eugene, OR). This mixture was then denatured by heating to 95°C for 10 min using a heat block. Subsequently, 20 µL of each sample was then loaded alongside an appropriate molecular weight marker (Mark12™ stained standard, Life Technologies) onto a 1 mm, 10 well 4-12% Bis-Tris protein gel (Life Technologies) and run for ~4 h at 10 V/cm in MOPS buffer. The completed gel was washed 3 times with H_2O, stained using SimplyBlue™ SafeStain (Life Technologies) for 1 h, and destained overnight in H_2O. The gel was then analyzed using an Odyssey Infrared Gel Scanner (Li-Cor Biosciences, Lincoln, NE).

Size Exclusion Chromatography

Size exclusion chromatography was performed on a Shimadzu UFLC HPLC system. 100 µg (0.67 nmol) of each construct was run on an SEC column (Superdex™ 200 Increase 10/300 GL, GE Healthcare) in phosphate buffered saline for 40 min (flow rate: 0.75 mL/min) and the absorbance was measured at 280 nm.

Flow Cytometry

Flow cytometry experiments were performed with HER2-positive BT474 cells. Native pertuzumab and the immunoconjugates were incubated at 6 µg/mL in suspension with 10^6 cells/mL for 30 min on ice. Cells were washed three times by pelleting and resuspension and then incubated with a goat anti-human IgG-AlexaFluor568 secondary antibody (Thermo Fisher Scientific) at 4 µg/mL. Subsequently, the cells were again washed by pelleting and resuspension three times and then analyzed on a BD LSR II (BD Biosciences, San Jose, CA). Binding data was collected in triplicate, averaged, and plotted.

FcγRI Binding ELISA

Recombinant human Fc gamma RI/CD64, CF (R&D Systems # 1257-FC-050) was diluted to 10 µg/mL in sterile PBS and 100 µL/well was coated overnight at 4°C onto an ELISA plate (Nunc MaxiSorp® flat-bottom 96 well plate, Fisher Scientific). After a brief blocking period (40 min with PBS containing 10% FCS), the immunoconjugates were diluted in a blocking buffer (0.5 or 50 µg/mL) and 100 µL/well were applied for 2 h at room temperature. As not to disrupt the Fc-FcγRI interactions, the bound immunoconjugates were detected using 1: 5000 HRP-labeled anti human IgG (JacksonImmunoResearch Laboratories, West Grove, PA). After a final wash step, TMB substrate was used to develop the bound HRP secondary antibody, and the color reaction was stopped with 2N H_2SO_4. Optical Densities at 450 nm were determined using a SpectraMax i3 plate reader (Molecular Devices, San Jose, CA). Binding data was collected in triplicate, averaged, and plotted.

Radiolabeling with ^{89}Zr

For each antibody construct, 400 µg (2.67 nmol) of immunoconjugate solution was diluted in 400 µL PBS, pH 7.4. [^{89}Zr]Zr-oxalate (1300 µCi, 48.1 MBq) in 100 µL of 1.0 M oxalic acid was adjusted to pH 7.0-7.5 with 1.0 M Na_2CO_3. After the bubbling of CO_2 stopped, the ^{89}Zr solution was added to the antibody solution, and the resulting mixture was incubated at room temperature for 1 h. The reaction progress was then assayed using iTLC using an eluent of 50 mM EDTA (pH 5). Subsequently, the reaction was quenched with 10 µL of 50mM of EDTA (pH = 5), and the antibody construct was purified using size exclusion chromatography (Sephadex G-25 M, PD-10 column, GE Healthcare; dead volume = 2.5 mL, eluted with 500 µL fractions of PBS, pH 7.4). If necessary, the antibody was concentrated via centrifugal filtration units with a 50,000 Da molecular weight cut off (Amicon™ Ultra 4 Centrifugal Filtration Units, Millipore Corp. Billerica, MA). The radiochemical purity of the final radiolabeled bioconjugate was assayed by iTLC using 50 mM EDTA (pH 5) as an eluent. In the iTLC experiments, free [^{89}Zr]Zr^{4+} cations and [^{89}Zr]-EDTA elute with the solvent front, while the radioimmunoconjugate remains at the baseline.

Bead-Based Immunoreactivity Assay

A bead-based assay was used to determine the immunoreactive fraction of each of the immunoconjugates. To this end, 20 μL of Ni-NTA beads (Thermo Fisher Scientific # 88831) were transferred to Eppendorf™ LoBind microcentrifuge tubes, and 380 μL of PBS-T (PBS with 0.05% Tween-20) was added. The tubes were vortexed for 5 sec, spun down, and placed on a DynaMag-2 magnetic rack (Invitrogen) for 30-45 sec. Once the beads settled, the supernatant was aspirated and discarded. The tubes were then placed on the magnetic rack, and 400 μL of PBS-T was added. 3 of the tubes were capped, labeled C_1-C_3, and set aside as controls.

Next, to prepare the antigen-coupled Ni-NTA beads, 1 μg (10 μL of 0.1 mg/mL dilution) of the His-tagged HER2 antigen (Acro Biosystems, Newark, DE) was added to the rest of the tubes (labeled T_1-T_3), and the tubes were placed on a rotating platform for 15 min. The tubes were then spun down and placed onto the magnetic rack, and the supernatant was aspirated. The same sequence of steps was repeated to wash the beads with 400 μL of PBS-T. Prior to addition of the radioimmunoconjugate in the next step, the beads were resuspended in 399 μL of 1% BSA-PBS.

Subsequently, each radioimmunoconjugates was diluted to 1 ng/μL with PBS, and 1 ng of the diluted radioimmunoconjugate was added to all the tubes. The tubes were vortexed for 5 seconds and placed on the rotating platform for 30 min. The tubes were once again spun down and placed on the magnetic. The supernatant and two washes (400 μL each) were collected. Finally, the beads, supernatant, and washes were measured for radioactivity on a gamma counter (Automatic Wizard^2 ɣ-counter, Perkin Elmer, Inc., Waltham, MA). The immunoreactive fraction was then calculated as the fraction of activity associated with the beads corrected for the fraction of non-specific binding observed in the control samples.

Radioimmunoconjugate Stability Assays

The stability of the radioimmunoconjugates with respect to radiochemical purity and loss of radioactivity from the antibody was investigated via incubation of the antibodies in human serum for 7 days at 37°C (n = 3). At predetermined time intervals, the radiochemical purity of the radioimmunoconjugates was determined via iTLC with an eluent of 50 mM EDTA pH 5.0.

PET Imaging

PET imaging experiments were conducted on a microPET Focus rodent scanner (Concorde Microsystems). Mice (athymic nude or humanized NSG mice) bearing subcutaneous BT474 xenografts (left shoulder, 60-120 mm^3, 25-30 days after inoculation) were administered the radioimmunoconjugates [175-210 μCi, 6.5-7.8 MBq (40-80 μg) in 200 μL of saline] via tail vein injection. Approximately 5 min before PET imaging, the mice were anesthetized by inhalation of a 2% isoflurane (Baxter Healthcare):oxygen gas mixture and placed on the scanner bed. Anesthesia was maintained using a 1% isoflurane mixture. PET data for each mouse were recorded via static scans at 24, 48, 96 and 144 h post-injection (n = 4-5 per group).

Acute Biodistribution Experiments

Mice (athymic nude or humanized NSG mice) bearing subcutaneous BT474 xenografts (left shoulder; 60-120 mm^3) were randomized before the study and were administered the radioimmunoconjugates [20 μCi, 0.74 MBq (10 μg) in 200 μL of saline] via tail vein injection. Subsequently, the animals (n = 5 per group) were euthanized by CO_2(g) asphyxiation at 24, 48, 96, and 144 h p.i., and 13 tissues (including the tumor) were removed, washed, dried, weighed, and counted in a gamma counter (PerkinElmer, Inc.). The number of counts in each tissue were background and decay corrected to the time of injection and converted to activity units (μCi) using a calibration curve generated from known standards. The %ID/g for each tissue sample was then calculated by normalization to the total activity injected and the mass of each tissue.

SUPPLEMENTAL FIGURES AND FIGURE CAPTIONS

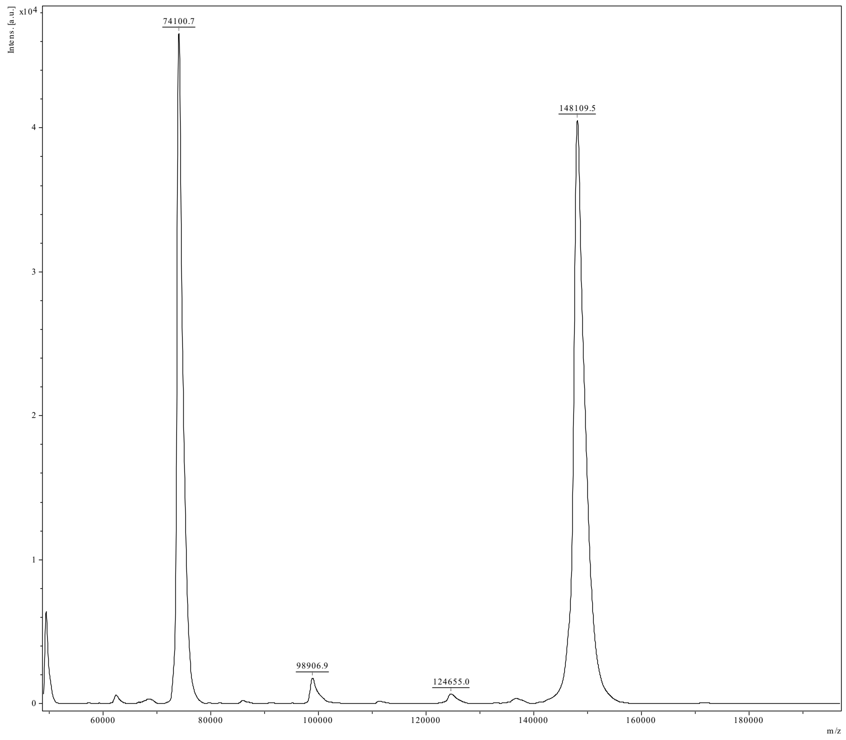

Figure S1. Representative MALDI-ToF spectrum for native pertuzumab.

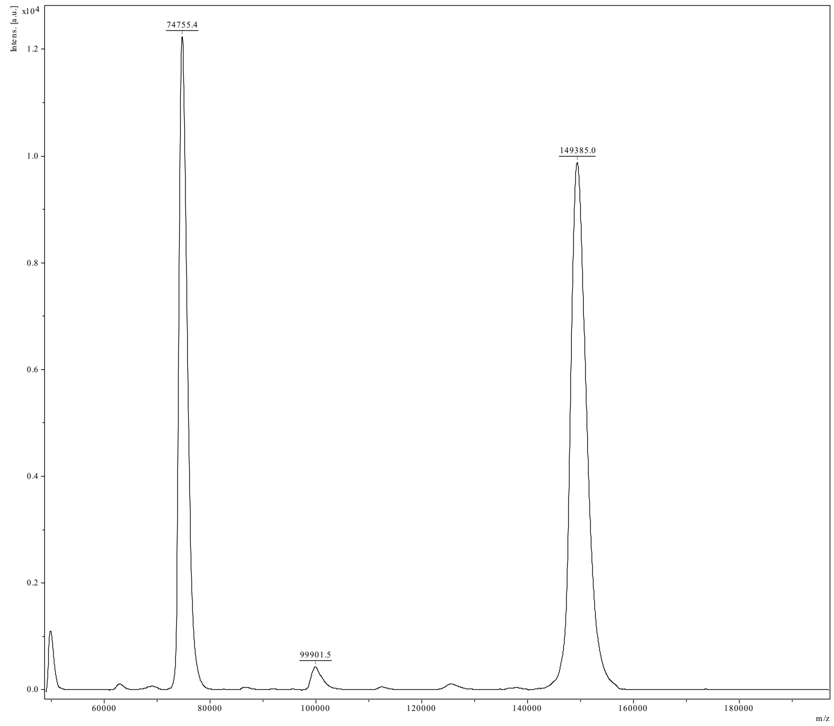

Figure S2. Representative MALDI-ToF spectrum for DFO-^{nss}pertuzumab.

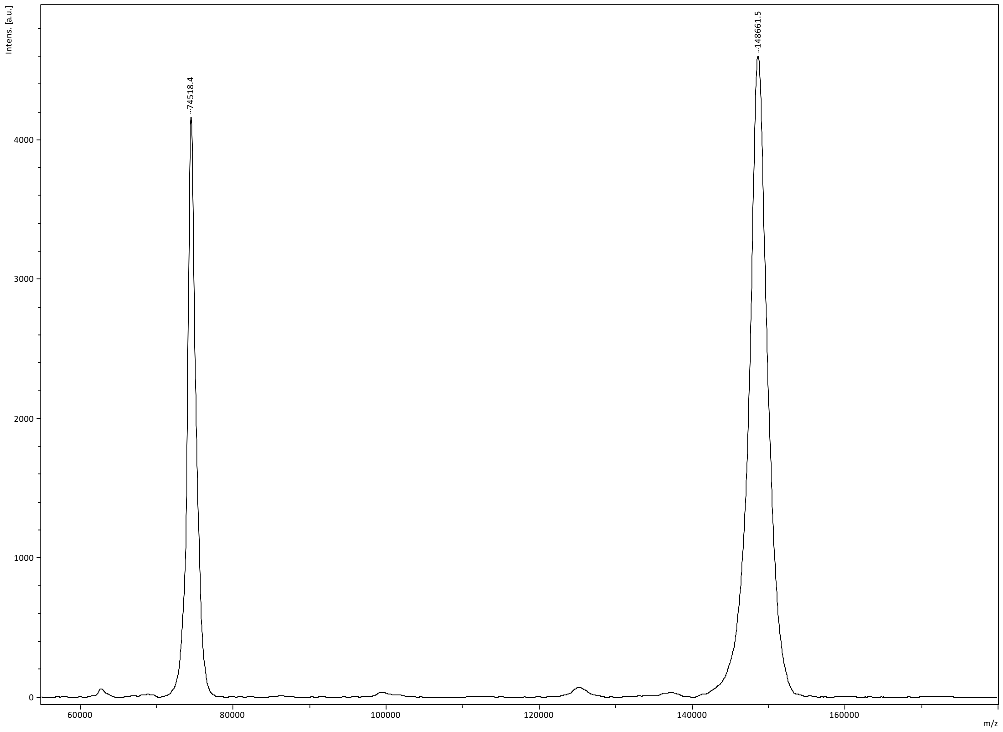

Figure S3. Representative MALDI-ToF spectrum for N_3-[ss]pertuzumab-βGal.

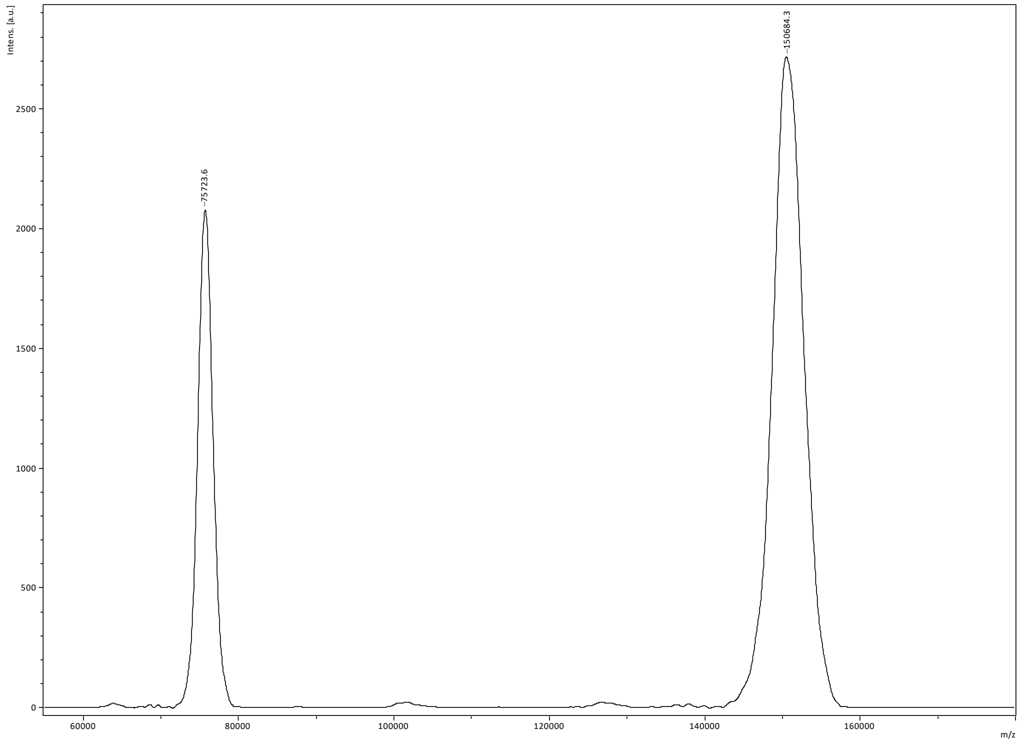

Figure S4. Representative MALDI-ToF spectrum for DFO-[ss]pertuzumab-βGal.

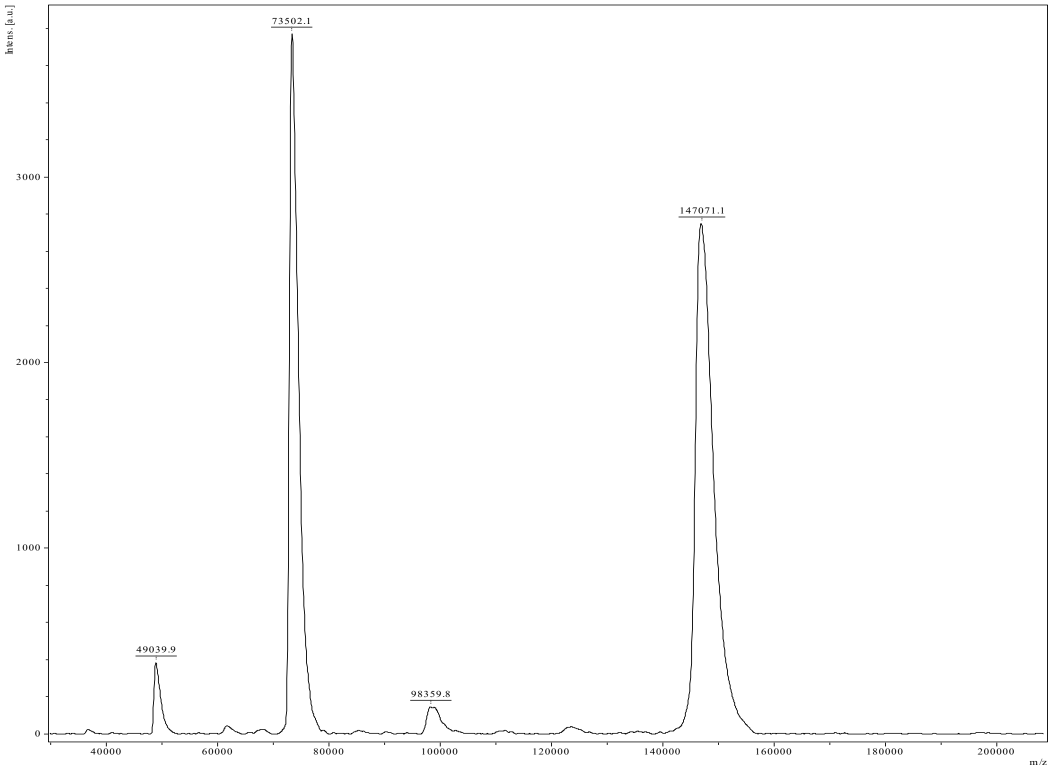

Figure S5. Representative MALDI-ToF spectrum for N_3-^{ss}pertuzumab-EndoS.

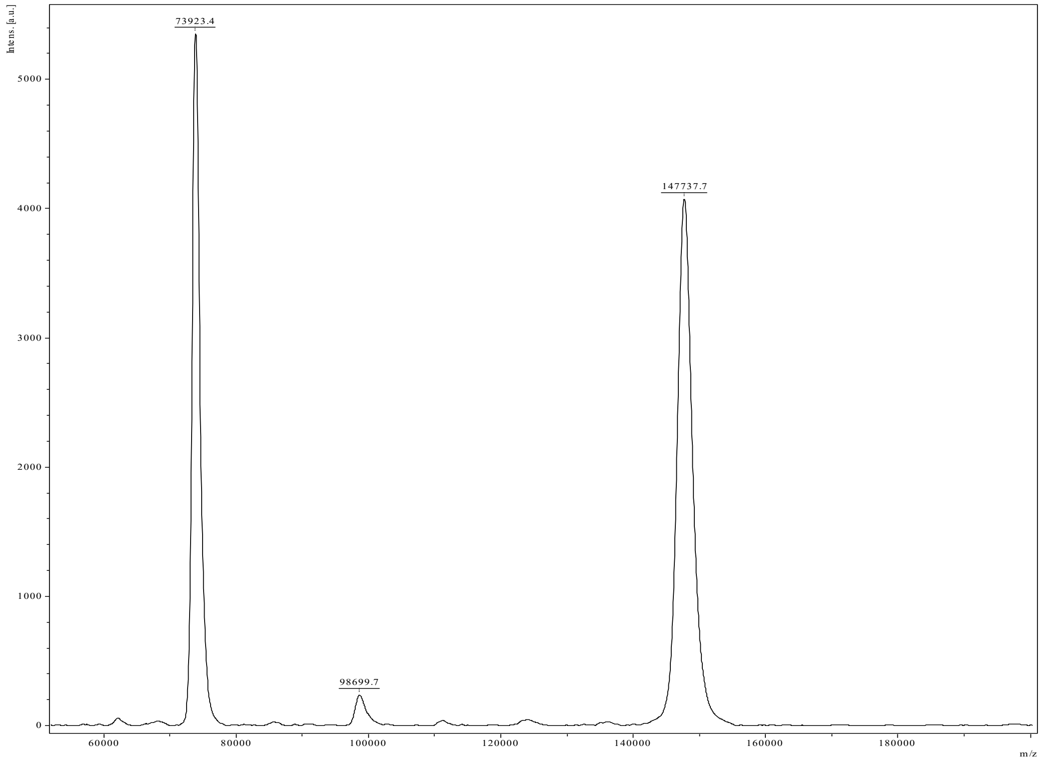

Figure S6. Representative MALDI-ToF spectrum for DFO-^{ss}pertuzumab-EndoS.

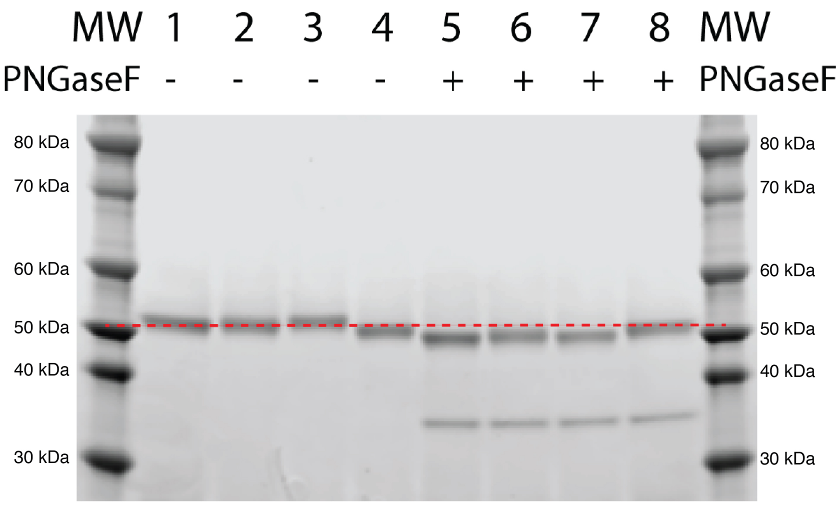

Figure S7. Denaturing SDS-PAGE of unmodified pertuzumab (1 & 5), DFO-^{nss}pertuzumab (2 & 6), DFO-^{ss}pertuzumab-βGal (3 & 7), and DFO-^{ss}pertuzumab-EndoS (4 & 8) before (1-4) and after (5-8) PNGaseF-mediated deglycosylation. After deglycosylation, the heavy chain of each of the immunoconjugates has the same molecular weight, except that DFO-^{ss}pertuzumab-EndoS (because PNGaseF is unable to digest the immunoconjugate's truncated glycans). The red dotted line denotes the mass of the heavy chain of native pertuzumab.

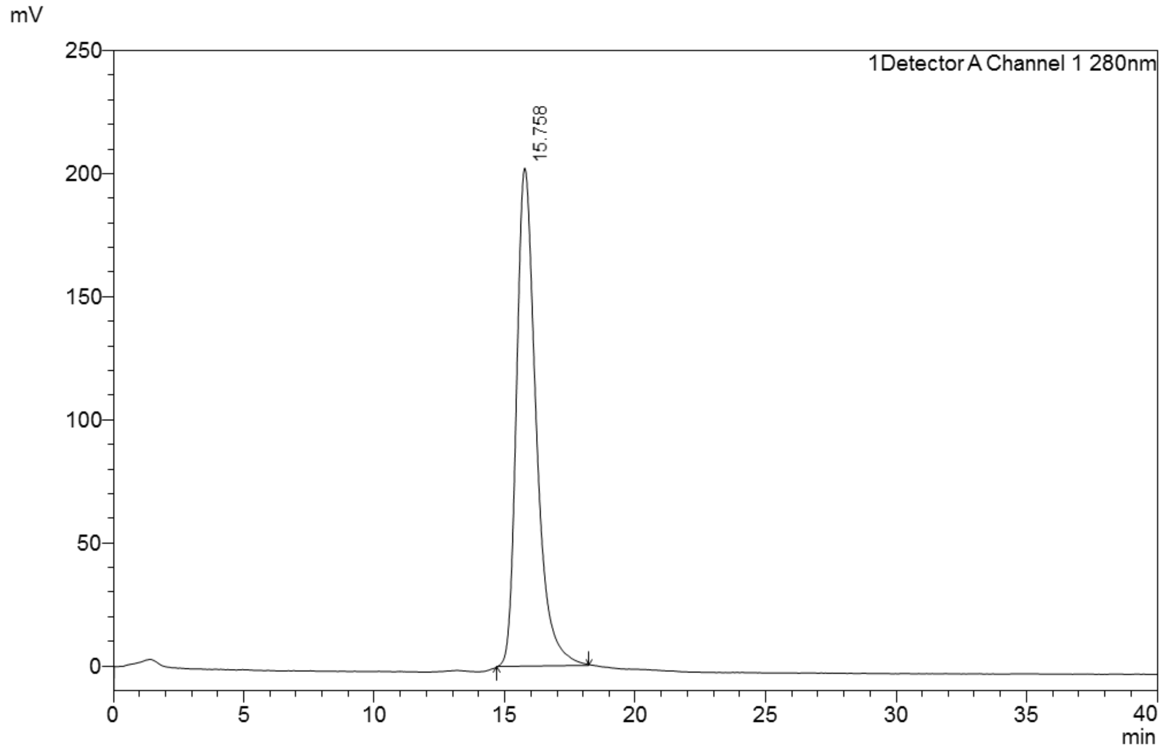

Figure S8. Size exclusion chromatogram of native pertuzumab

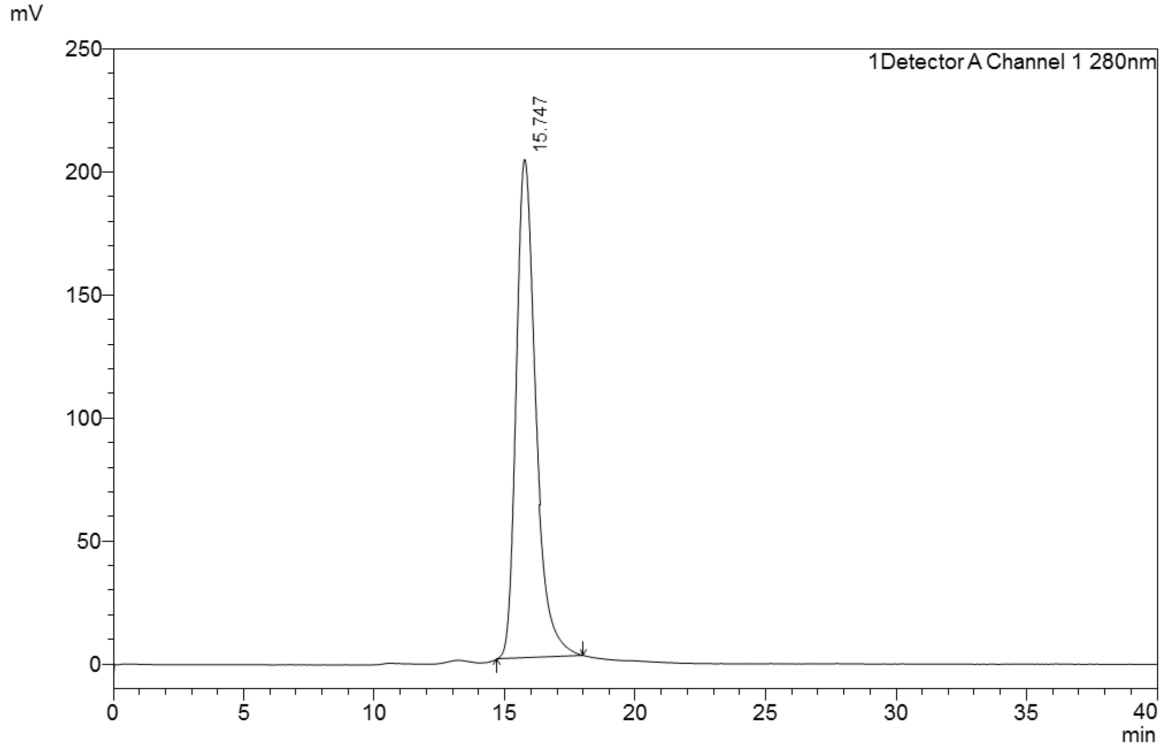

Figure S9. Size exclusion chromatogram of DFO-^{nss}pertuzumab.

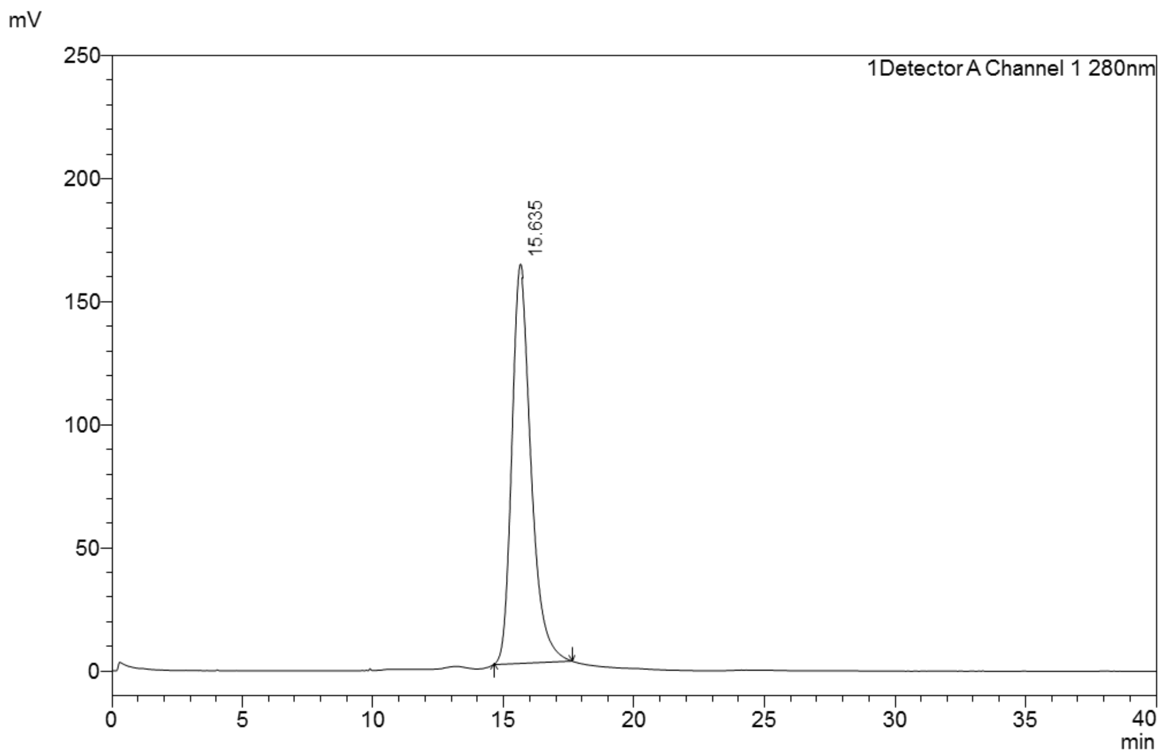

Figure S10. Size exclusion chromatogram of DFO-[ss]pertuzumab-βGal.

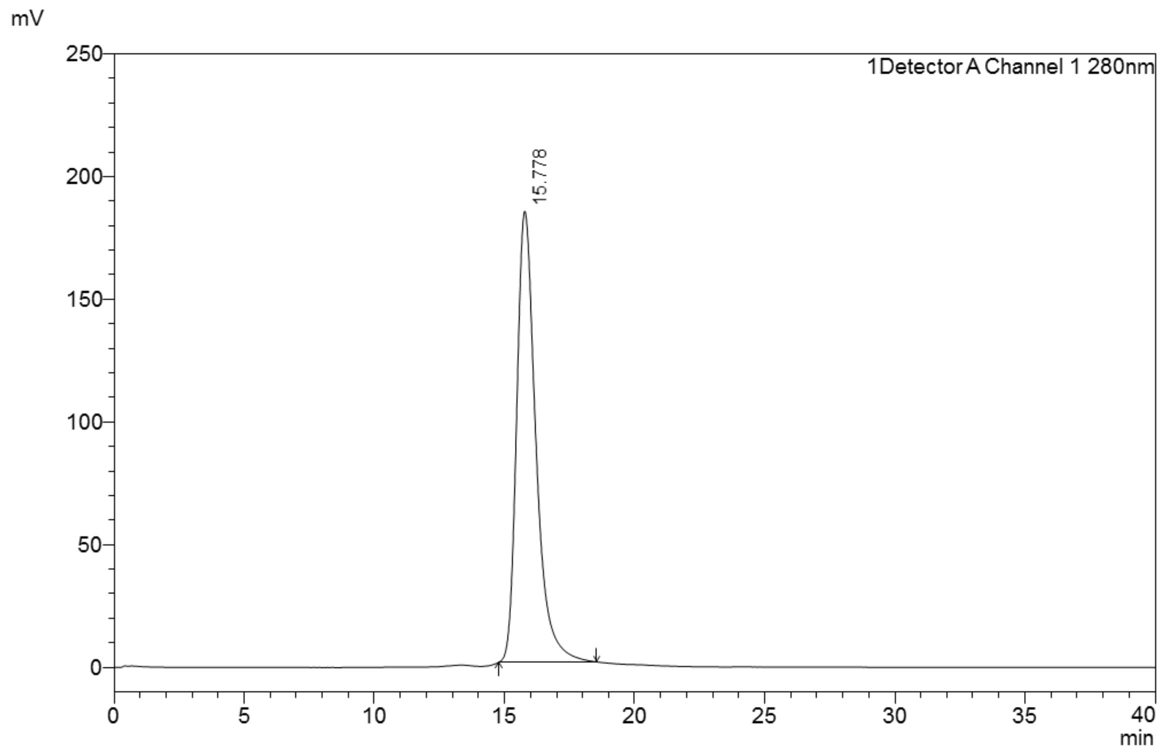

Figure S11. Size exclusion chromatogram of DFO-[ss]pertuzumab-EndoS.

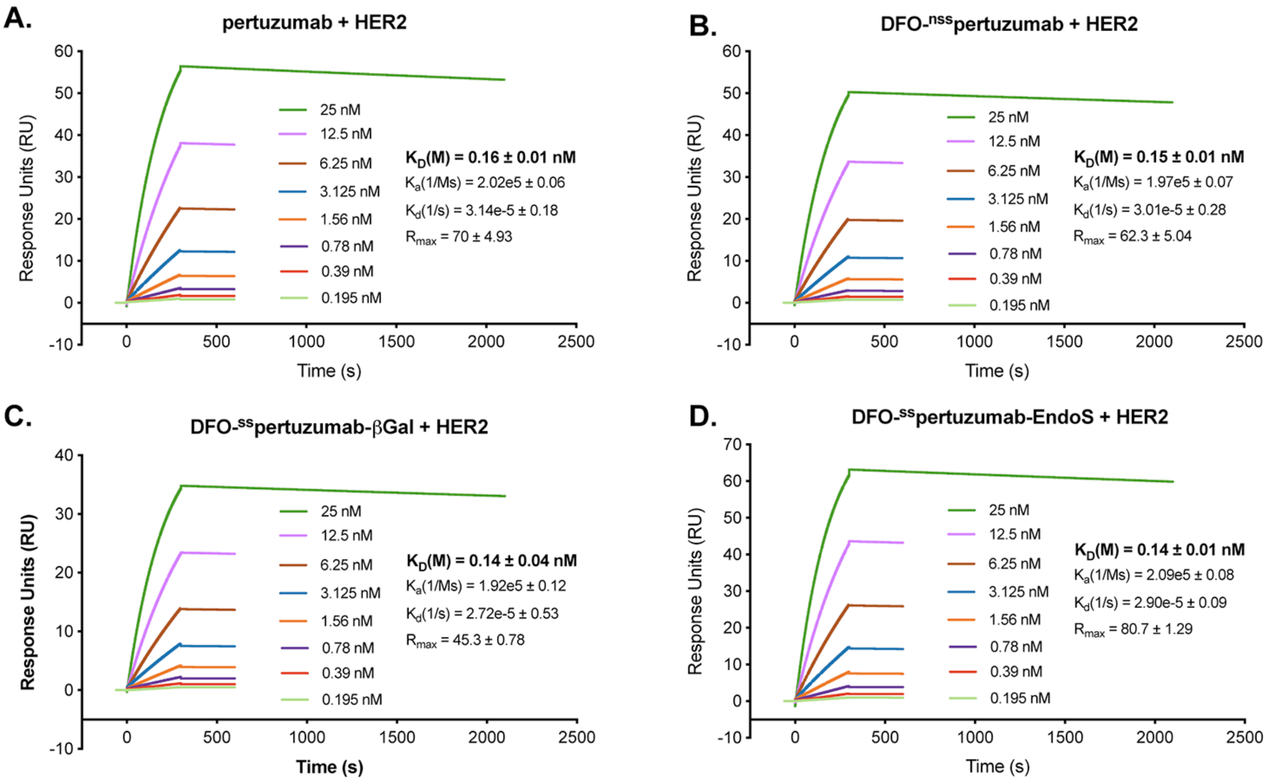

Figure S12. SPR sensorgrams for the binding of (A) native pertuzumab, (B) DFO-^{nss}pertuzumab, (C) DFO-^{ss}pertuzumab-EndoS, and (D) DFO-^{ss}pertuzumab-βGal to recombinant HER2.

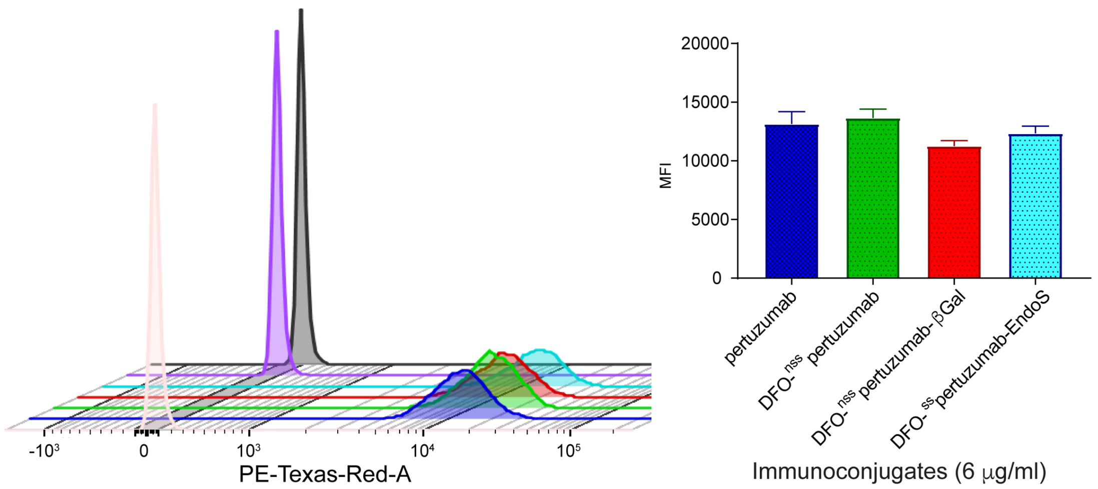

Figure S13. Flow cytometry data showing the binding of the immunoconjugates to HER2-expressing BT474 human breast cancer cells.

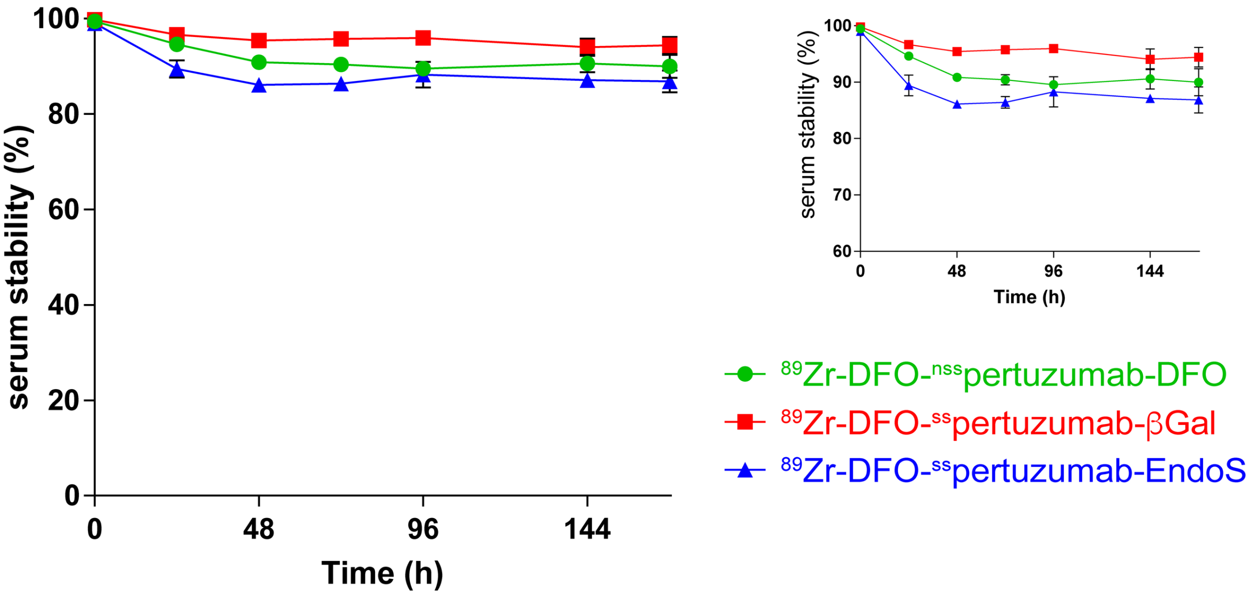

Figure S14. Stability of the three radioimmunoconjugates to demetallation in human serum at 37°C. Measurements were collected using radio-iTLC and performed in triplicate.

SUPPLEMENTAL TABLES AND TABLE LEGENDS

Constructs	Average mass (Da)	Degree of Labeling (DFO/mAb)
pertuzumab	148264 ± 110	1.4 ± 0.4
DFO-^{nss}pertuzumab	149300 ± 185	
N_3-^{ss}pertuzumab-βGal	148645 ± 12	2.6 ± 0.1
DFO-^{ss}pertuzumab-βGal	150574 ± 86	
N_3-^{ss}pertuzumab-EndoS	147145 ± 59	1.3 ± 0.2
DFO-^{ss}pertuzumab-EndoS	146998 ± 60	

Table S1. Degree of labeling of each immunoconjugate as determined via MALDI-MS.

	^{89}Zr-DFO-^{nss}pertuzumab				^{89}Zr-DFO-^{ss}pertuzumab-βGal				^{89}Zr-DFO-^{ss}pertuzumab-EndoS			
	**24 h**	**48 h**	**96 h**	**144 h**	**24 h**	**48 h**	**96 h**	**144 h**	**24 h**	**48 h**	**96 h**	**144 h**
**Blood**	9.22 ± 0.72	7.17 ± 2.68	5.42 ± 2.16	4.88 ± 2.09	10.41 ± 0.95	8.18 ± 3.29	7.86 ± 1.36	5.77 ± 1.53	9.84 ± 0.84	8.33 ± 3.31	6.12 ± 2.11	4.70 ± 2.12
**Tumor**	30.99 ± 1.84	45.36 ± 10.54	41.30 ± 10.54	93.15 ± 52.16	31.60 ± 2.10	47.88 ± 14.39	61.05 ± 10.67	77.15 ± 32.99	35.60 ± 9.12	54.64 ± 18.38	64.43 ± 23.38	76.68 ± 28.67
**Heart**	3.11 ± 0.85	2.84 ± 1.11	1.89 ± 0.71	1.90 ± 0.80	3.88 ± 0.75	2.77 ± 1.18	2.75 ± 0.94	1.86 ± 0.30	3.39 ± 0.47	3.02 ± 1.30	2.12 ± 0.77	1.78 ± 0.79
**Lung**	4.40 ± 0.65	3.65 ± 1.04	3.06 ± 1.01	2.90 ± 0.98	4.09 ± 0.55	3.79 ± 1.53	4.36 ± 0.88	3.72 ± 0.97	4.13 ± 0.65	4.14 ± 1.61	3.55 ± 1.10	2.84 ± 1.21
**Liver**	3.37 ± 0.50	3.86 ± 1.76	4.10 ± 1.19	4.50 ± 2.38	2.51 ± 0.33	3.56 ± 1.26	4.92 ± 2.21	5.22 ± 2.04	1.96 ± 0.08	2.43 ± 0.65	2.47 ± 1.02	2.47 ± 1.17
**Spleen**	2.04 ± 0.34	2.41 ± 1.05	1.96 ± 0.97	1.68 ± 0.53	1.71 ± 0.21	2.11 ± 0.65	1.94 ± 0.59	1.99 ± 0.43	1.87 ± 0.14	1.91 ± 0.62	1.75 ± 0.34	1.77 ± 0.53
**Stomach**	1.35 ± 0.71	0.77 ± 0.22	0.63 ± 0.22	0.45 ± 0.18	0.79 ± 0.35	0.78 ± 0.44	0.62 ± 0.16	0.53 ± 0.15	0.70 ± 0.27	0.90 ± 0.31	0.67 ± 0.38	0.54 ± 0.27
**SI**	1.21 ± 0.19	1.22 ± 0.51	0.88 ± 0.42	0.63 ± 0.27	1.11 ± 0.20	0.98 ± 0.36	1.03 ± 0.19	0.63 ± 0.05	1.13 ± 0.11	1.03 ± 0.35	0.78 ± 0.24	0.63 ± 0.32
**LI**	1.47 ± 0.31	1.40 ± 0.39	0.96 ± 0.43	0.69 ± 0.15	1.02 ± 0.47	0.90 ± 0.20	0.82 ± 0.07	0.66 ± 0.16	0.99 ± 0.71	0.73 ± 0.22	0.92 ± 0.22	0.48 ± 0.15
**Kidney**	3.74 ± 0.12	3.52 ± 0.64	3.56 ± 0.59	3.47 ± 0.85	3.81 ± 0.35	4.10 ± 0.90	5.40 ± 0.54	5.72 ± 1.21	3.63 ± 0.41	4.49 ± 1.16	4.25 ± 1.24	4.86 ± 1.57
**Muscle**	0.81 ± 0.41	0.90 ± 0.32	0.60 ± 0.14	0.60 ± 0.21	1.08 ± 0.11	0.76 ± 0.21	0.84 ± 0.29	0.71 ± 0.34	1.08 ± 0.31	0.91 ± 0.23	0.51 ± 0.14	0.49 ± 0.16
**Bone**	1.87 ± 0.48	3.94 ± 3.32	5.25 ± 3.39	8.42 ± 2.36	1.28 ± 0.41	1.78 ± 0.50	2.24 ± 0.44	2.68 ± 0.66	1.81 ± 0.60	3.00 ± 0.69	4.06 ± 1.32	6.50 ± 0.96
**Skin**	3.73 ± 0.37	4.44 ± 0.55	3.15 ± 1.07	2.55 ± 0.94	4.64 ± 0.59	4.51 ± 1.97	4.78 ± 1.58	5.38 ± 2.56	3.77 ± 0.66	5.06 ± 2.37	3.59 ± 2.50	4.09 ± 3.00

Table S2. Biodistribution data for the three constructs in nude mice bearing BT474 subcutaneous xenografts. The values are %ID/g ± SD; n = 5 mice for each time point and radioimmunoconjugate. SI = small intestine; LI = large intestine. The stomach, SI, and LI values include contents.

	^{89}Zr-DFO-^{nss}pertuzumab				^{89}Zr-DFO-^{ss}pertuzumab-βGal				^{89}Zr-DFO-^{ss}pertuzumab-EndoS			
**Tumor/**	**24 h**	**48 h**	**96 h**	**144 h**	**24 h**	**48 h**	**96 h**	**144 h**	**24 h**	**48 h**	**96 h**	**144 h**
**Blood**	3.36 ± 0.33	6.32 ± 2.78	7.62 ± 3.61	19.10 ± 13.47	3.04 ± 0.34	5.86 ± 2.94	7.76 ± 1.91	13.37 ± 6.73	3.62 ± 0.98	6.56 ± 3.42	10.52 ± 5.27	16.31 ± 9.55
**Heart**	9.97 ± 2.79	15.95 ± 7.24	21.84 ± 9.95	49.05 ± 34.41	8.14 ± 1.67	17.30 ± 9.02	22.19 ± 8.50	41.58 ± 19.02	10.51 ± 3.06	18.10 ± 9.88	30.43 ± 15.62	43.06 ± 24.95
**Lung**	7.05 ± 1.12	12.44 ± 4.56	13.50 ± 5.65	32.14 ± 21.04	7.73 ± 1.16	12.65 ± 6.38	14.01 ± 3.75	20.73 ± 10.39	8.62 ± 2.59	13.20 ± 6.80	18.17 ± 8.69	27.04 ± 16.66
**Liver**	9.19 ± 1.48	11.75 ± 6.02	10.07 ± 3.89	20.68 ± 15.94	12.60 ± 1.85	13.46 ± 6.26	12.40 ± 5.98	14.79 ± 8.58	18.20 ± 4.73	22.51 ± 9.66	26.05 ± 14.27	31.02 ± 19.78
**Spleen**	15.17 ± 2.67	18.79 ± 9.28	21.05 ± 11.71	55.54 ± 35.63	18.47 ± 2.59	22.66 ± 9.77	31.53 ± 11.12	38.71 ± 18.51	19.08 ± 5.08	28.55 ± 13.29	36.71 ± 15.14	43.33 ± 24.22
**Stomach**	22.98 ± 12.14	58.67 ± 21.81	66.01 ± 28.60	206.49 ± 142.31	40.16 ± 17.87	61.42 ± 39.57	98.09 ± 30.03	146.09 ± 75.09	51.06 ± 23.73	60.40 ± 29.09	95.78 ± 64.52	143.29 ± 84.49
**SI**	25.69 ± 4.26	37.31 ± 17.98	46.97 ± 25.34	147.32 ± 103.77	28.43 ± 5.54	48.68 ± 22.90	59.25 ± 14.88	121.94 ± 53.13	31.63 ± 8.66	52.87 ± 25.39	82.89 ± 39.71	120.89 ± 86.03
**LI**	21.15 ± 4.66	32.33 ± 11.70	42.93 ± 22.25	135.04 ± 81.33	31.09 ± 14.45	52.97 ± 19.72	74.19 ± 14.36	117.47 ± 58.16	35.97 ± 27.37	74.96 ± 34.16	70.36 ± 30.47	161.07 ± 94.37
**Kidney**	8.28 ± 0.56	12.90 ± 3.80	11.59 ± 3.53	26.84 ± 16.41	8.30 ± 0.95	11.67 ± 4.34	11.30 ± 2.27	13.49 ± 6.44	9.79 ± 2.74	12.16 ± 5.15	15.15 ± 7.06	15.78 ± 7.06
**Muscle**	38.25 ± 19.36	50.43 ± 21.37	69.04 ± 24.18	156.35 ± 104.07	29.13 ± 3.64	63.04 ± 25.70	72.85 ± 28.14	109.22 ± 70.84	32.99 ± 12.79	59.73 ± 24.99	126.32 ± 57.76	157.71 ± 71.93
**Bone**	16.55 ± 4.32	11.51 ± 10.07	7.86 ± 5.46	11.06 ± 6.93	24.72 ± 8.06	26.93 ± 11.11	27.31 ± 7.23	28.75 ± 14.17	19.71 ± 8.29	18.24 ± 7.42	15.86 ± 7.71	11.81 ± 4.18
**Skin**	8.31 ± 0.95	10.22 ± 2.70	13.11 ± 5.58	36.52 ± 24.46	6.81 ± 0.97	10.61 ± 5.62	12.78 ± 4.78	14.34 ± 9.18	9.44 ± 2.93	10.79 ± 6.23	17.97 ± 14.14	18.75 ± 14.03

Table S3. Tumor-to-tissue activity concentration ratios for the three constructs in nude mice bearing BT474 subcutaneous xenografts. The values are %ID/g ± SD; n = 5 mice for each time point and radioimmunoconjugate. SI = small intestine; LI = large intestine. The stomach, SI, and LI values include contents.

	^{89}Zr-DFO-^{nss}pertuzumab	^{89}Zr-DFO-^{ss}pertuzumab-βGal	^{89}Zr-DFO-^{ss}pertuzumab-EndoS
	144 h	*144 h*	*144 h*
**Blood**	1.03 ± 0.63	4.51 ± 3.00	8.76 ± 2.31
**Tumor**	46.13 ± 16.46	77.53 ± 12.56	111.84 ± 39.88
**Heart**	0.90 ± 0.04	1.76 ± 0.78	2.56 ± 0.23
**Lung**	2.40 ± 0.50	4.41 ± 1.49	6.55 ± 1.07
**Liver**	10.25 ± 2.74	8.87 ± 3.53	4.71 ± 0.77
**Spleen**	44.77 ± 7.92	22.39 ± 17.37	13.09 ± 3.99
**Stomach**	0.74 ± 0.22	1.08 ± 0.78	0.88 ± 0.25
**SI**	2.69 ± 0.53	2.44 ± 0.62	1.99 ± 0.49
**LI**	1.00 ± 0.07	0.92 ± 0.44	1.20 ± 0.73
**Kidney**	3.94 ± 1.97	5.44 ± 0.65	4.43 ± 0.72
**Muscle**	0.63 ± 0.17	0.69 ± 0.11	0.90 ± 0.18
**Bone**	9.14 ± 3.05	5.84 ± 2.11	7.24 ± 2.04
**Skin**	2.44 ± 0.29	5.34 ± 3.57	4.64 ± 2.20

Table S4. Biodistribution data for the three radioimmunoconjugates at 144 h post-injection in humanized NSG mice bearing BT474 subcutaneous xenografts. The values are %ID/g ± SD; n = 4 mice for ^{89}Zr-DFO-^{nss}pertuzumab, n = 5 mice for ^{89}Zr-DFO-^{ss}pertuzumab-βGal, and n = 5 mice for ^{89}Zr-DFO-^{ss}pertuzumab-EndoS. SI = small intestine; LI = large intestine. The stomach, SI, and LI values include contents.

	^{89}Zr-DFO-^{nss}pertuzumab	^{89}Zr-DFO-^{ss}pertuzumab-βGal	^{89}Zr-DFO-^{ss}pertuzumab-EndoS
Tumor/	*144 h*	*144 h*	*144 h*
**Blood**	44.91± 19.91	17.21 ± 11.78	12.77 ± 5.66
**Heart**	51.30 ± 18.42	43.95 ± 20.71	43.73 ± 16.08
**Lung**	19.21 ± 7.93	17.57 ± 6.58	17.09 ± 6.70
**Liver**	4.50 ± 2.01	8.74 ± 3.75	23.72 ± 9.30
**Spleen**	1.03 ± 0.41	3.46 ± 2.75	8.54 ± 4.01
**Stomach**	62.14 ± 28.82	71.89 ± 53.04	127.34 ± 57.69
**SI**	17.13 ± 6.98	31.84 ± 9.60	56.26 ± 24.41
**LI**	46.08 ± 16.79	84.15 ± 42.52	93.44 ± 65.84
**Kidney**	11.72 ± 7.20	14.26 ± 2.87	25.23 ± 9.90
**Muscle**	73.33 ± 32.53	112.22 ± 25.95	124.41 ± 50.67
**Bone**	5.05 ± 2.47	13.28 ± 5.27	15.45 ± 7.02
**Skin**	18.90 ± 7.11	14.53 ± 10.01	24.12 ± 14.33

Table S5. Tumor-to-tissue activity concentration ratios for the three radioimmunoconjugates at 144 h post-injection in humanized NSG mice bearing BT474 subcutaneous xenografts. The values are %ID/g ± SD; n = 4 mice for ^{89}Zr-DFO-^{nss}pertuzumab, n = 5 mice for ^{89}Zr-DFO-^{ss}pertuzumab-βGal, and n = 5 mice for ^{89}Zr-DFO-^{ss}pertuzumab-EndoS. SI = small intestine; LI = large intestine. The stomach, SI, and LI values include contents.
